# Compositional Phase Control in High-Entropy Alloy Electrocatalysts

**DOI:** 10.1021/jacs.5c16422

**Published:** 2026-01-22

**Authors:** Sangmin Jeong, Anthony J. Branco, Porvajja Nagarajan, Connor S. Sullivan, Ji Hyeon Cha, Silas W. Bollen, Noah L. Mason, Milinda Abeykoon, Daniel Olds, Michael B. Ross

**Affiliations:** † Department of Chemistry, 14710 University of Massachusetts Lowell, Lowell, Massachusetts 01854, United States; ‡ National Synchrotron Light Source II, 8099 Brookhaven National Laboratory, Upton, New York 11973, United States

## Abstract

High-entropy alloys (HEAs) provide uniquely tunable structural and electronic properties that enable robust electrocatalysis. While compositional manipulation of HEAs is well-known, systematically controlling the crystalline phase and morphology remains a challenge that could provide new avenues for controlling reactive sites and physical properties. Here, we show the preferential stabilization of mixed fcc/bcc to fcc phases by controlling the Au content in quinary AuPdFeCoNi HEA nanoparticles. This systematic structural and compositional control, when investigated with an ensemble of electronic, X-ray synchrotron, and surface techniques, allows us to identify the critical short- (few-Å) and medium- (6–10 Å) range structural motifs that deliver exceptional hydrogen evolution reaction (HER) catalysis. Specifically, these HEAs exhibit both outstanding durability (240 h) and high mass activity (50 A/mg_PGM_) normalized to noble metal content, outperforming commercial Pt/C (3.18 A/mg_PGM_). This structural control over HEA morphology, and its direct association with changes in specific metallic oxidation states and pair–pair atomic structural features, provides new means and strategies for finely designing robust and sustainable electrocatalysts with a majority nonprecious metal composition.

## Graphical abstract

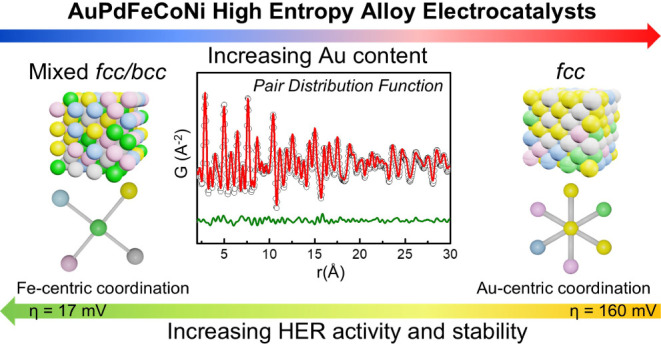

## Introduction

High-entropy alloys (HEAs) have remarkable stability, strength, hardness, and corrosion resistance afforded by their entropically stabilized physical crystalline structures.^1,2^ Recently, HEAs have attracted attention for tunable electrocatalytic activity and durability because their electronic structures can be finely tuned by an almost infinitely wide combinatorial set of 5 or more elements.^3–5^ As such, the local structures and electronic properties of highly stable HEA interfaces have the potential to overcome the limitations of conventional electrocatalysts.^6,7^ While electronic tunability over composition has been well-studied, precise control over the combined compositional and structural phase is less well developed. Precise control over the phase allows one to control active sites in ways that are challenging or impossible in conventional enthalpically stabilized alloys.

Control of crystalline phase and structure in HEAs has shown potential for electrochemical applications, including energy production,^8–10^ storage,^11–13^ and fuel cells^14–16^ using lower concentrations of platinum-group metals (PGMs) (e.g., platinum (Pt), iridium (Ir), palladium (Pd), and ruthenium (Ru)) due to their unique alloy design space and enhanced activity sites. However, the development of HEA nanoparticles presents several challenges related to their phase stability, atomic fractions, and uniformity. The complex elemental compositions also make it difficult to predict the synergistic effects during the alloying process, especially under varying environmental conditions such as temperature, atomic vacancies, and preferential bonding of certain elements. As a result, most of the reported studies on HEAs are limited to a single structure, such as face-centered cubic (fcc),^10,14,17^ body-centered cubic (bcc),^18–20^ orhexagonal close-packed (hcp).^7,21,22^ Consequently, achieving systematic control over phase and confirming their structures remains a challenge for analysis due to the compositional and structural complexity of HEAs. Accordingly, a sustained commitment to advancing our understanding of HEA structures and analyzing reaction mechanisms is critical.

By combining the synchrotron X-ray pair distribution function (PDF) analysis with simulation results based on theoretical models, it is possible to determine the particle size and interpret complex metal nanostructures in HEAs. This approach shows key features such as local lattice distortion, atomic structures, and stress-induced atomic-level rearrangements, as previously reported in synchrotron X-ray studies of HEAs.^23–26^ Importantly, the PDF is derived from the Fourier transform of the total scattering data, offering a direct representation of interatomic distance probabilities within the sample. It is a well-established technique, aided by high-energy synchrotron X-ray radiation thousands of times more intense than conventional hard X-rays, enabling precise atomic-level characterization of complex systems and providing key insights into atomic arrangements and interatomic distances.^23–28^ PDF analysis has been widely used to characterize energy-related technologies such as sodium-ion batteries^29–31^ and fuel cells.^32^ For HEAs, it allows one to analyze complex structures over multiple nanometers, providing a powerful approach to link structural motifs with catalytic performance. By linking structural motifs to catalytic performance, this allows one to advance catalyst design over multiple length scales, bridging local coordination environments and long-range order.^33–35^

Herein, we show that Au-containing HEA nanoparticles can have controlled phase behavior from a body-centered cubic (bcc) to a face-centered cubic (fcc) structure. Specifically, AuPdFeCoNi HEA nanoparticles were synthesized using thermally assisted wet chemical reduction.^36^ Structurally, they are characterized using high-resolution electron microscopy and synchrotron-based X-ray total scattering. Electronically, cyclic voltammetry and X-ray photoelectron spectroscopy were used to understand their electrochemical oxidation–reduction properties and the chemical states of the alloyed metallic elements. Meanwhile, based on the compositionally controlled phase in HEAs, ranging from mixed fcc/bcc to fcc, we observed significant electrochemical hydrogen evolution activity with a low overpotential (17 mV vs RHE, @10 mA/cm^2^) and 15.7 times enhanced mass activity, along with exceptional durability—more than 18 times higher than that of Pt/C (wt. 10%) in acidic electrolytes.

## Results and Discussion

AuPdFeCoNi HEAs were synthesized by dissolving Au, Pd, Fe, Co, and Ni metal chloride precursors in a preheated aqueous solution, with the heating mantle set to maintain 230 °C. Then, poly­(*N*-vinyl-2-pyrrolidone) (PVP, M.W. ≈ 40,000) was added to the solution. After that, upon formation of a black solid suspension, HEA nanoparticles were collected by centrifugation. A detailed synthesis procedure is provided in the Supporting Information. This strategy revealed that the control of phase from mixed fcc/bcc to fcc can be controlled as a function of Au content in the HEA (Figure 1a).

**Figure 1:**
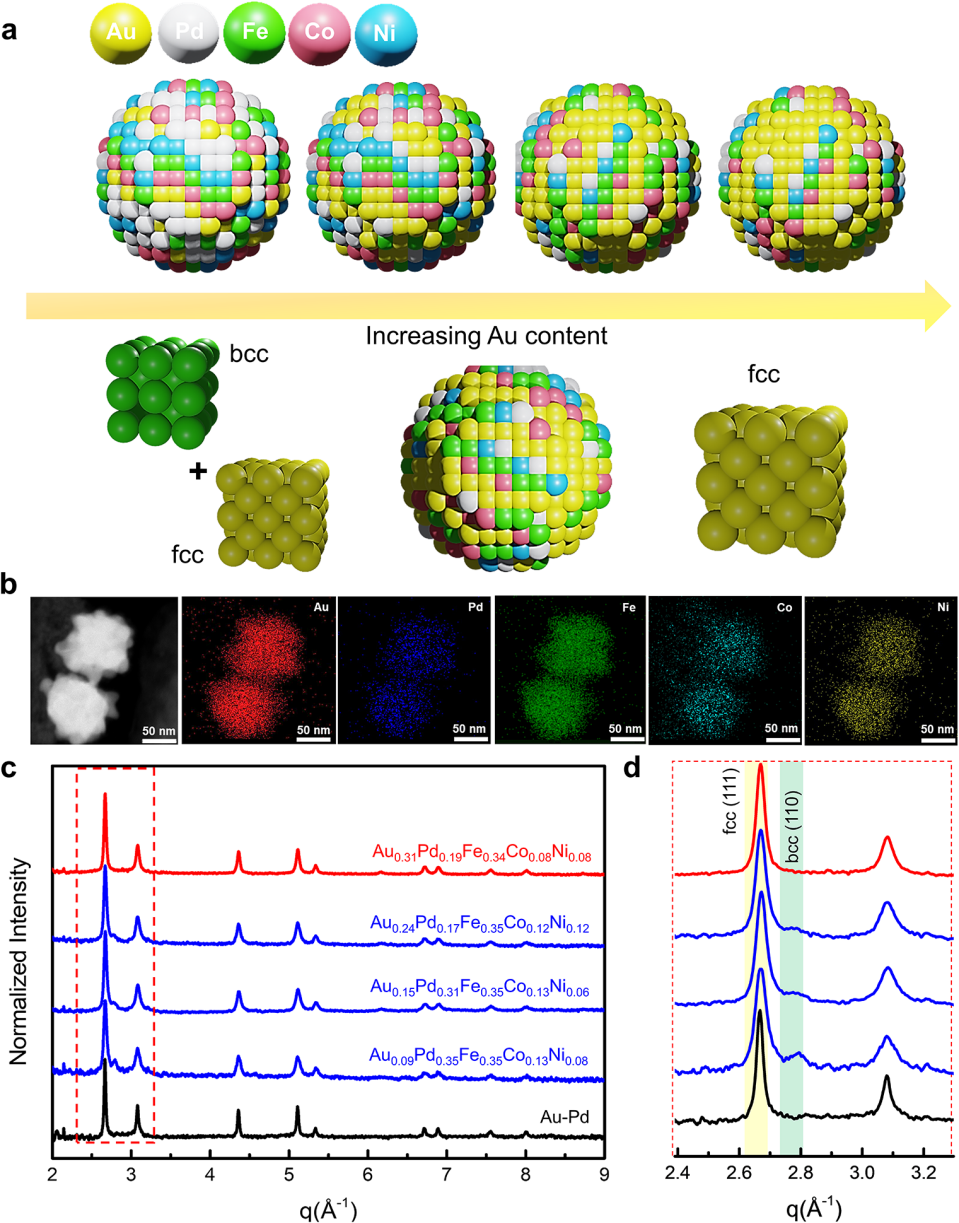
Schematic representation of the transformation in crystal structure from mixed fcc/bcc to fcc with increasing Au contents. (b) STEM images accompanied by the corresponding EDS mapping for AuPdFeCoNi HEAs. (c) Synchrotron-based wide-angle X-ray scattering (WAXS) spectra of AuPdFeCoNi HEAs. The fitting for HEAs with 9–24 at. % Au (blue) and 31 at. % Au (red) is shown. (d) An enlarged view of the WAXS profiles around the (111) and (110) reflections.

The phase control and evolution of the various AuPdFeCoNi HEAs were investigated by synchrotron-based wide-angle X-ray scattering (WAXS). As a first approximation of phase content, the (111) [for fcc at 2.67 Å^–1^] and (110) [for bcc at 2.80 Å^–1^] Bragg reflections were analyzed (Figure 1c,d). The initially synthesized AuPdFeCoNi HEAs displayed a mixed fcc/bcc phase. However, we found that increasing Au content can stabilize a single fcc phase, the AuPdFeCoNi HEA in the fcc phase. This is confirmed by the change in the intensities of the (111) and (110) reflections. In all cases, the (110) bcc reflection decreased with increasing Au content, eventually disappearing (low-Au content, mixed fcc/bcc indicated by blue; high-Au content, fcc indicated by red). These are confirmed in-house using powder X-ray diffraction (XRD) (Figure S1). Both diffraction techniques revealed identical phase control characteristics, suggesting that the phase control and atomic arrangements of HEAs can be controlled by manipulating the relative Au composition. The elemental composition of the HEAs was quantified by inductively coupled plasma optical emission spectroscopy (ICP-OES), revealing that the HEAs contained Au, Pd, Fe, Co, and Ni in the range of 5–35 at. % (Table S1).

To better understand the nanoscale structure and crystallite morphology, high-resolution electron microscopy was used. Dark-field scanning transmission electron microscopy (DF-STEM) and energy-dispersive X-ray spectroscopy (EDS) elemental mapping images of the synthesized HEAs (Figure 1b, Figures S2 and S3) show a uniform distribution of individual metals of Au, Pd, Fe, Co, and Ni across the nanoparticles. High-resolution TEM, fast Fourier transform (FFT), and selected-area electron diffraction (SAED) analysis show the prototypical atomic arrangement of the bcc phase (ABAB planar lattice stacking arrangements) and the fcc phase (ABCABC stacking arrangements)^37–41^ in the synthesized AuPdFeCoNi HEAs (Figure 2). Additional EDS spectra with line scan profiles and selected-area electron diffraction (SAED) were performed to complement the FFT-based structural analysis (Figure S3). In all cases, we do not see significant segregation of the integrated elements; they appear well mixed.

**Figure 2:**
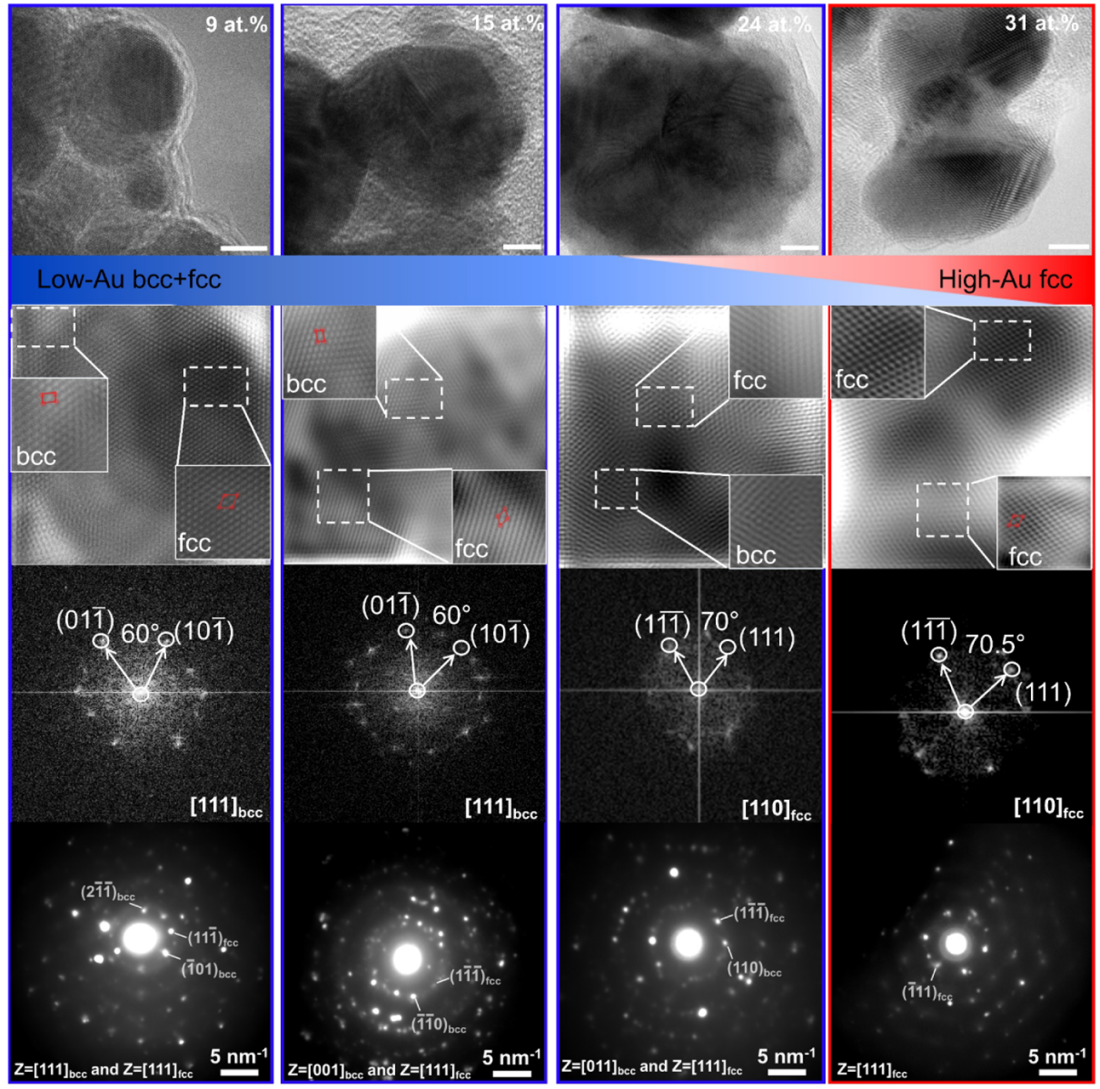
Bcc-to-fcc phase manipulation in AuPdFeCoNi HEAs. HR-TEM, lattice- and atomic-resolved HR-TEM, fast Fourier transform (FFT), and selected-area electron diffraction (SAED) images of AuPdFeCoNi HEA bcc and fcc structural motifs. The blue boxes indicate bcc-rich regions, and the red boxes indicate fcc phase regions (scale bar: 5 nm).

We observe that changing Au content changes the fcc content; this behavior is dependent on the specific metal concentration, which governs the stabilized phases, as theoretically predicted previously.^42^ For example, Fe has been reported to favor the formation of the bcc-phase alloy structures.^37^ Correspondingly, for lower Au contents (9–24 at. %, blue), both bcc and fcc phases are observed by FFT and SAED (Figure 2 and Figure S3). The different rings and spot patterns correspond to the (110), (200), (211), and (310) planes, while the spot patterns indicate a mixture of bcc and fcc reflections with rectangular arrays in HEA with 9–24 at. % Au. In contrast, at higher Au content (31 at. %, red), only the fcc phase is observed, with the diffraction rings corresponding to the (111), (200), (220), and (311) planes, and the spot pattern exhibiting hexagonal symmetry. These results indicate that the Au content dictates the phase, with higher Au concentrations preferentially stabilizing the fcc structure.

Understanding the atomic arrangements in HEA nanostructures requires multiple characterization approaches to understand the integrated structural and electronic properties.^24,26,43^ Synchrotron pair distribution function (PDF) analysis was used to systematically characterize the local ordering behavior and chemical bonding motifs in fcc/bcc mixed-phase AuPdFeCoNi HEA nanoparticles. Unlike conventional X-ray diffraction tools that primarily assess long-range order, PDF analysis provides both long-range atomic arrangements (over 10 Å) and detailed short-range structural information, including bond lengths and local defects.^26,44^

Bimetallic, ternary, and quaternary alloy models were constructed to reveal the pair–pair bond correlations in the putative HEA alloys (Figure 3a, Figures S4–S7). The partial pair correlation functions for specific local nearest-neighbor pairs (e.g., Au–Fe, Au–Ni, Au–Co) were analyzed using *PDFgui*.^44^ Several clear structural motifs emerge, including local distortions in the structure near ∼4.5 Å, changes in coordination environments, and broadening effects (e.g., 4.75–5.2 Å) that are indicative of the configurational disorder characteristic of HEAs. Notably, the first peak (2.86–2.88 Å) of the experimental AuPdFeCoNi sample closely matches those of the simulated alloys, indicating a consistent short-range atomic arrangement dominated by heavy elements, such as Au and Pd.^45,46^ As anticipated, HEAs with higher Au contents were better fit (*R*_wp_ decreased from 21 to 12%) by an fcc model compared to the lower Au content structures, which have mixed fcc/bcc phases (Figure 3b, Figures S6–S8). Medium-range correlations below ∼5 Å are found to be associated with Au–Fe, Au–Co, and Au–Ni interactions (Figure 3c), while features extending beyond 5 Å are dominated by longer-range Au–Pd correlations. Additionally, Pd–Fe interactions induce a structural distortion around 8.1 Å, whereas Ni–Ni and Ni–Pd interactions contribute to distortions at 4.56 and 4.9 Å.

**Figure 3:**
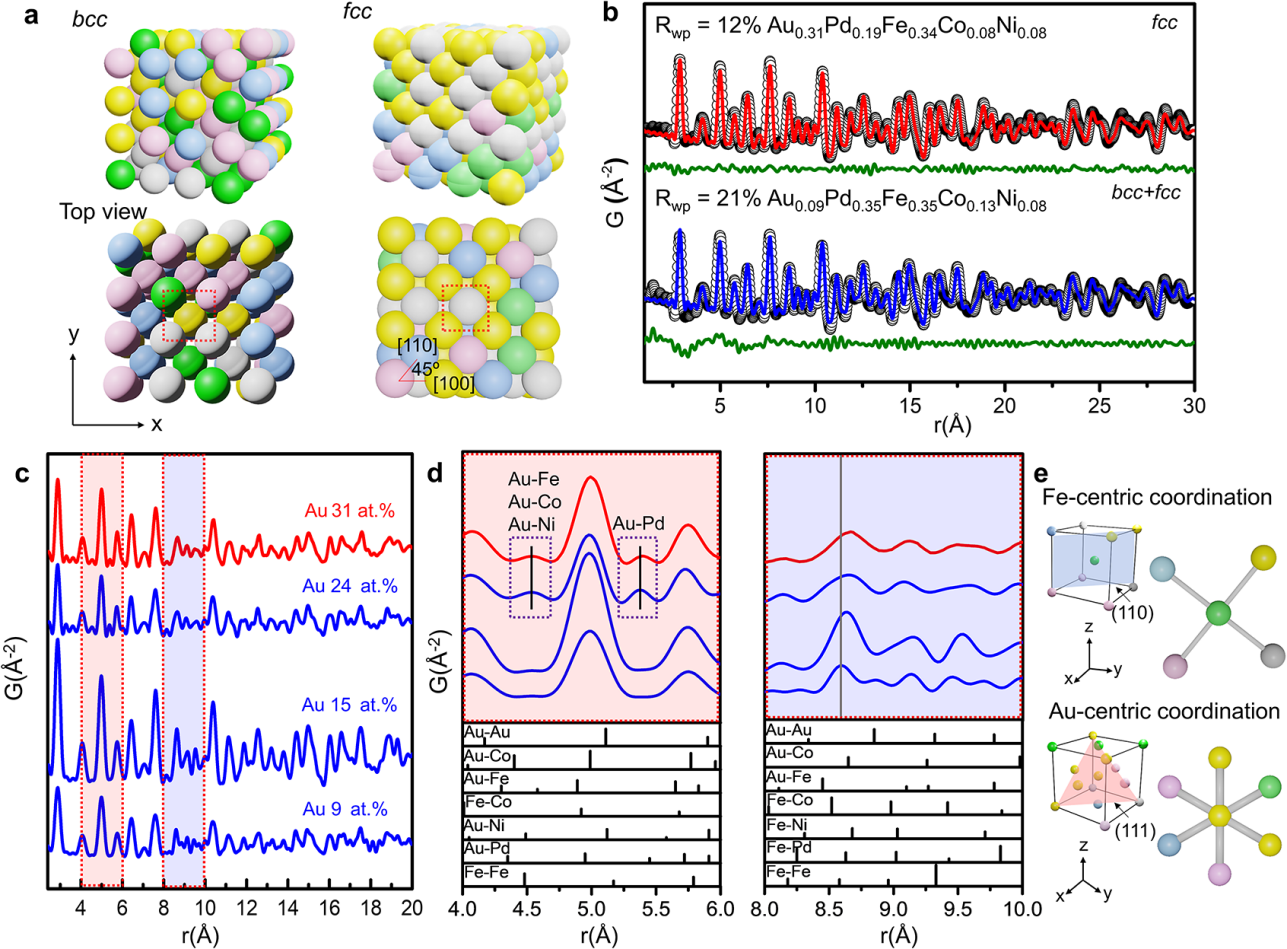
Local structure characterization and simulation of AuPdFeCoNi HEAs. (a) Schematic illustrations of HEA structures with bcc and fcc, (b) the experimental PDF and simulated PDF data for HEAs with 9 at. % Au (*R*_wp_ = 21%) and 31 at. % Au (*R*_wp_ = 12%), (c) comparison of the observed PDF data for HEAs with different Au contents, (d) enlarged views of the PDF profiles around the peaks at *r* ∼6.0 and ∼10.0 Å, highlighting certain nearest-neighbor pairs and corresponding theoretical partial pair correlation functions, and (e) schematic of the lattice structure in bcc and fcc HEAs.

Figure 3c,d and Figures S5–S6 show that the Au–Au pair distance decreases from 5.1 to 5.0 Å, while the distances for pairs involving Au, such as Au–Pd, Au–Fe, Au–Ni, and Au–Co pair distances, increase monotonically from 8.58 to 8.68 Å with increasing Au content. These changes suggest local structural rearrangements and lattice distortion induced by the compositional mixing within the HEA. The large atomic radius of Au compared to Fe, Ni, and Co contributes to asymmetric expansion in certain local environments, while complex atomic interactions and packing effects result in pair–pair contraction in others.^47,48^ Specifically, the atomic size mismatch between Au and other constituent metals, along with variations in their electronic configurations and bonding characteristics, results in local lattice distortions manifesting as contraction or expansion of atomic distances within the alloy.^45,46^ Additionally, as noted above, the EDS analysis does not suggest significant elemental segregation within these systems. Figure 3e illustrates the 3D atomic arrangements and characteristic crystallographic planes of the bcc and fcc lattices, providing visual insight into the observed lattice distortions and the structural transition toward a predominant fcc phase with an increasing Au content.

To complement the physical structural insights from PDF and HR-TEM, XPS was employed to quantify the composition-dependent electronic changes in the HEA nanoparticles. The Au 4f, Pd 3d, and 2p orbitals of Fe, Ni, and Co were characterized by XPS to determine the metallic state and surface composition in HEA systems (Figure S9). With an increase in Au incorporation, a positive shift of the Au 4f_7/2_ binding energy (BE) from 83.6 to 84 eV was measured. Positive shifts were also observed in the Pd 3d and Fe 2p spectra, which shifted by 0.2–0.5 eV toward higher binding energies in the high-Au content alloys compared to the low-Au content alloys (Figure S9 and Table S2). However, for Co 2p and Ni 2p, a negative shift in the binding energies was observed, with Co 2p shifting from 780.6 to 780.5 eV and Ni 2p shifting from 854.5 to 854.0 eV with increasing Au content. Specifically, the broadening of Co 2p, accompanied by a strong satellite feature, indicates oxidation. As the Au content increased, Co 2p showed an increased fraction of higher oxidation states, reflective of partial reduction of Co while retaining some Co^2+^ state.^49–53^ In contrast, Ni 2p exhibited a decrease in both binding energy and satellite peak intensity, indicative of charge redistribution and a lower oxidation state.^54,55^ These results demonstrate that the oxidation states of Ni and Co are modified differently compared to those of Au and Pd in the HEA system, likely due to their distinct local chemical environments. The observed shifts in the binding energies of the Au 4f, Pd 3d, Fe 2p, Ni 2p, and Co 2p orbitals are consistent with previously reported binary, ternary, and more complex multimetallic systems and have been attributed to the formation of Au-based HEAs at the atomic level.^56–59^

With structural and electronic characterization in hand, we then sought to understand how the diverse and tunable structures of AuPdFeCoNi HEAs impact their electrocatalytic properties toward the hydrogen evolution reaction (HER). Historically, Au has not been a focus of HER research due to its high activation energy requirements and low intrinsic activity in the pure phase.^60^ However, we have previously found Au-containing HEAs have reasonable activity across a range of pHs.^36^ Therefore, considering the unique structure and catalytic durability of systematically controlled HEAs, we sought to quantify the HER capabilities of these HEA nanoparticles with tunable phases. HER activities of four HEA electrocatalysts were measured and compared with commercial Pt/C (10 wt. %). With the exception of the 31 at. % Au HEA, AuPdFeCoNi HEAs exhibit HER catalytic activities similar to commercial Pt/C, with lower onset potentials and overpotentials along with remarkable durability in extended testing (Figure 4).

**Figure 4:**
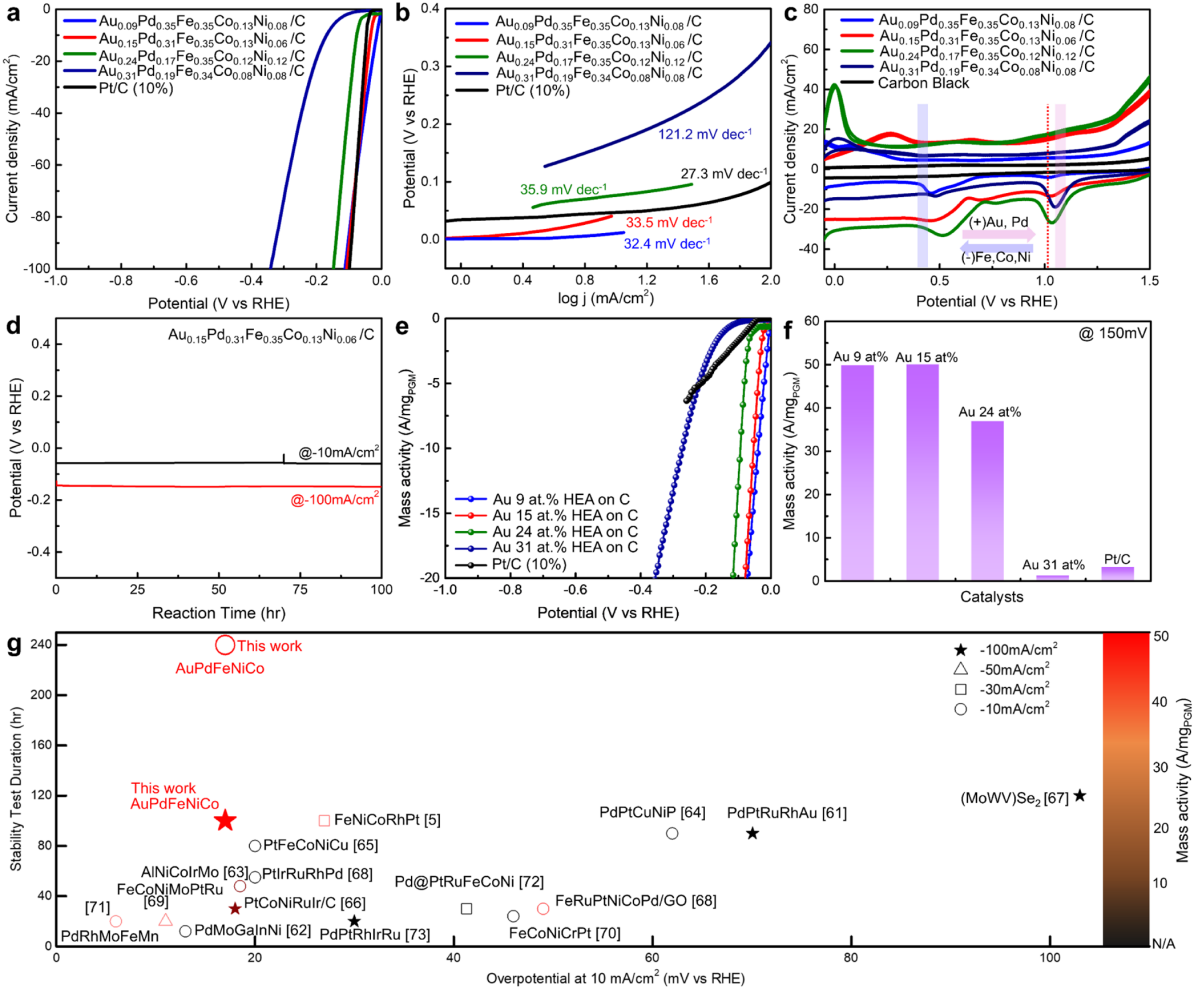
Evaluation of electrochemical HER activity in phase controlled HEAs. (a) HER polarization curves of the as-synthesized catalysts, (b) corresponding Tafel slope, (c) comparison of the reduction–oxidation values of AuPdFeCoNi HEAs in the potential range of −0.1 V to 1.5 V, (d) long-term stability of HEAs (15 at. % Au) for 100 h at 10 mA/cm^2^ and 100 mA/cm^2^, (e) mass activity polarization of the as-synthesized catalysts, and (f) mass activity at 150 mV. (g) Comparison of HER activities with recently reported HEA catalysts in 0.5 M H_2_SO_4_ electrolyte.^61–73^

The electrochemical HER for the phase-controlled HEA electrocatalysts required varying overpotentials to achieve a current density of 10 mA/cm^2^. Specifically, the overpotentials were Au 9 at. % (17 mV), Au 15 at. % (32 mV), Au 24 at. % (78 mV), and Au 31 at. % (160 mV), as shown in Figure 4a. Tafel slopes were determined for AuPdFeCoNi HEAs and Pt/C to assess the kinetic properties of the electrocatalysts, with HEA (Au 9 at. %, 32.4 mV dec^–1^), HEA (Au 15 at. %, 33.5 mV dec^–1^), HEA (Au 24 at. %, 35.9 mV dec^–1^), HEA (Au 31 at. %, 121.2 mV dec^–1^), and Pt/C (27.3 mV dec^–1^) reflecting desirable kinetic electrochemical activity (Figure 4b). At higher Au content (31 at. %), the HER activity decreased substantially, likely due to the poor electrocatalytic properties of Au hindering proton adsorption.^5^

Building on the Tafel slope analysis, the electrochemical redox behavior of HEAs with different compositions was investigated by using cyclic voltammetry (CV). Figure 4c shows that the CV of the Au-enriched HEAs exhibits a significant shift to a more positive potential in the negative scan direction. This is attributed to the intrinsic electronic properties of Au,^74^ which has a higher reduction potential compared to the other constituent metals (Pd,^75^ Fe,^76^ Co,^77^ and Ni^78^). This behavior indicates that the phase character and electronic interactions of other metals in HEA modulate the electrochemical activity at the catalyst-substrate interface. Specifically, as the Au content increases, a monotonic shift in the first reduction peak from 1.0 to 1.05 V is observed. This correlates with a reduced catalytic activity for the HER, as evidenced by an increase in overpotential from 75 mV to 160 mV at 10 mA/cm^2^. Ultimately, this observation confirms the presence of metallic binding effects on the surface of the HEAs, which influence HER activity through the combined effects of electronic structure modifications, surface active sites, and atomic-scale interactions unique to the HEA composition.

The durability of HEAs was investigated using chronopotentiometry in a 0.5 M H_2_SO_4_ electrolyte. The potential of HEAs remained stable under both conditions (10 mA/cm^2^ for 10 days and 100 mA/cm^2^ for 100 h, respectively) (Figure 4d and Figures S10–S14), whereas Pt/C (10%) showed an increase in potential from 165 to 240 mV (vs RHE, retention 68.8%) over 100 h. Postdurability XRD analysis further confirmed that the structural integrity of the HEAs remained intact (Figure S13), underscoring their exceptional chemical and structural robustness under acidic conditions. The mass activity normalized to the loading of noble metals for HEA (Au 15 at. %) at a potential of −150 mV was 50, which was 15.7 times higher than that of commercial Pt/C (3.18 A/mg_PGM_) (Figure 4e,f). These results indicate that the economic cost of HEAs ($18.36/g) is lower than that of commercial Pt/C cathodes ($59.30/g, Sigma-Aldrich Chemical Co., Ltd.) (Table S3), suggesting their great potential in electrochemical hydrogen production applications.

Compared with previous studies, our HEAs demonstrate markedly improved HER activity in an acidic electrolyte (Figure 4g and Table S4). Notably, they even surpass noble-metal-only HEAs such as PdPtRuRhAu,^61^ PtIrRuRhPd,^68^ and PdPtRhIrRu^73^ in performance, despite containing a lower total noble metal content. This combination of high mass activity, low overpotential, and excellent durability positions our HEAs as among the most effective HER catalysts to date. These findings highlight the effectiveness of the multimetallic design strategy, which enables the synergistic enhancement of both catalytic activity and long-term stability.

By integration of the structural and spectroscopic information with catalytic data, the critical motifs can be understood, and their relative impacts can be distinguished. Synchrotron-PDF modeling shows that the most pronounced local Au–Pd interactions occur with increasing Au content, alongside significant atomic rearrangements also being observed, with Pd–Fe and Pd–Ni interactions contributing to longer-range few-Å distortions. Associated changes in the electronic environment are observed by XPS binding energy shifts, indicating an internal charge redistribution and altered electronic interactions. The observed shift in the first CV reduction peak aligns with these electronic modifications, further linking the phase mixture to an improved HER activity. Specifically, the highest HER activities were observed for Au 9 and 15 at. %, where mixed fcc/bcc to fcc structures are present. This suggests that the mixed structure, observed in the FFT (Figure 2) and synchrotron PDF modeling (Figure 3 and Figure S8), significantly influences the electronic properties, as seen in Figures S15 and S16. For Au, Pd, and Fe, increasing active metal oxidation state ratios, such as Au^1+^/Au^0^, Pd^2+^/Pd^0^, and Fe^3+^/Fe^2+^, correlated with higher overpotentials, which is consistent with the general trend that higher oxidation states of these metals tend to reduce electron transfer efficiency for the HER process.^77,78^ In contrast, for Ni and Co, higher ratios of satellite Ni/Ni^2+^ and satellite Co/Co^3+^ were associated with lower overpotentials, indicating that these oxidation states facilitate electron transfer and enhance catalytic activity.^79,80^

## Conclusion

We have shown that manipulating HEA composition and crystalline structure together can have pronounced impacts on catalytic activity toward HER such that precious metal content can be reduced, while mass activity and durability can be enhanced. Phase control in AuPdFeCoNi HEA nanoparticles was achieved by manipulating the Au content to create a series of mixed bcc/fcc phase and pure fcc structures. High-resolution electron microscopy and synchrotron X-ray scattering techniques verify the crystalline and local structure characteristics that govern the phase control. Moreover, AuPdFeCoNi HEAs exhibit exceptional electrocatalytic activity with remarkable durability for HER in acidic electrolyte, maintaining stable performance for 240 h with low overpotentials and high precious metal mass activity. It remains unclear what the primary driver of the phase preferences from bcc to fcc is. Higher-resolution electron microscopy could aid in understanding homogeneity structure at the sub-nm level. Alongside this, theoretical modeling would aid both in understanding the relative energetics of the preferred crystalline phases and in relating them to the catalytic activity. This work shows that the combination of these five elements forms a robust approach to understanding the structure of HEAs. This offers a viable alternative to platinum-group metal catalysts, extending the application of entropically stabilized electrocatalysts for electrochemical hydrogen production and other renewable energy conversions. We believe that the multielement approach of HEAs will inspire further advancements in sustainable energy conversion technologies.

## Supplementary Material



## Notes

Experimental and characterization details; XRD, STEM-EDS, STEM-SAED, Synchrotron-PDF, *PDFgui* simulation models of the local structure in bimetallic and AuPdFeCoNi HEAs, ICP-OES, and XPS analyses, electrochemical measurements, LSV analysis, and chronopotentiometry analysis (PDF)
