# Supplementary material for: Compositional Phase Control in High-Entropy Alloy Electrocatalysts
Source: J Am Chem Soc. 2026 Jan 22;148(5):5146–54. doi: 10.1021/jacs.5c16422 (PMC12903856; doi:10.1021/jacs.5c16422)
Supplement: Supplementary file 1 [file ja5c16422_si_001.pdf]

Supporting Information for:

Compositional Phase Control in High Entropy Alloy Electrocatalysts

Sangmin Jeong[1], Anthony J. Branco[1], Porvajja Nagarajan[1], Connor S. Sullivan[1], Ji Hyeon Cha[1], Silas W. Bollen[1], Noah L. Mason[1], Milinda Abeykoon,[2] Daniel Olds[2], Michael B. Ross[1,*]

[1]Department of Chemistry, University of Massachusetts Lowell, Lowell, MA 01854

[2]National Synchrotron Light Source II, Brookhaven National Laboratory, Upton, NY, USA, 11973

Email: michael_ross@uml.edu

Experimental

Chemicals.

The metal precursors of $AuCl_3$ (99.99%), $CoCl_2H_{12}O_6$ (99.998%), $NiCl_2$ (99.995%), and poly(N-vinyl-2-pyrrolidone) (PVP, M.W. 40,000) were purchased from Alfa Aesar (USA). $PdCl_2$ (99.99%), $FeCl_2H_8O_4$ (99%), ethanol, Nafion (5 wt.%), and Pt/C (10 wt.%) were purchased from Thermo Scientific (USA). The isopropyl alcohol (IPA) and vulcanized carbon (Carbon Black, XC-72) were purchased from Ricca and Fuel Cell Earth, respectively. Sulfuric acid (ACS reagent, 98%) purchased from Sigma-Aldrich was used to prepare the 0.5M H_2SO_4 electrolyte. All experiments used ultrapure water (18.2 MΩ). For the standard calibration curve used in the ICP-OES analysis, multi-element solution 3 (for Au and Pd, 10 μg/mL) and multi-element calibration standard 2A (for Fe, Co, and Ni, 10 μg/mL) from Agilent were used.

Synthesis of AuPdFeCoNi HEAs

The wet-chemical method was adopted for the synthesis of AuPdFeCoNi HEAs, as previously reported.[1] First, a mixture of equal molar amounts (0.5 mmol) of four metal precursors, excluding gold, and a controlled molar amount of gold (0.1, 0.5, 1, and 5 mmol) were completely dissolved in mixture of ethanol and DI water (3:1, total volume: 40 mL). To achieve homogeneous mixing, each metal precursor was sequentially added to the solution at 30-minute intervals, starting with gold and followed by the other metals. Then, the mixed solution was heated to 230°C in a vertical coiled condenser system to control the wetting process. Next, PVP was added to the preheated mixed metal solution. After 1 hour, when the solution turned black, it was allowed to cool to room temperature (RT, 20°C). Next, the obtained precipitate was washed five times with ethanol and DI water using centrifugation at 14,000 rpm for 15 min to remove residual PVP. Finally, the black

powder was thoroughly dried under vacuum. All sample preparation procedures are carried out in the same method.

Characterization of Materials

The general characterization of morphology and structure was obtained using a high-resolution, dark-field scanning transmission electron microscope (HR-TEM/DF-STEM, JEOL, JEM-2100Plus, 200 kV) and energy dispersive X-ray spectroscopy (EDS) elemental mapping. The fast Fourier transform (FFT) and the inverse FFT (IFFT) patterns were performed using Gatan and Image J software. Selected-area electron diffraction (SAED) were also performed to complement the FFT-based structural analysis. X-ray diffraction (XRD) was measured using a Rigaku Miniflex X-ray diffractometer (CuKα radiation, λ = 1.5406 Å) measurements. X-ray photoelectron spectroscopy (XPS) was performed using a PHI Versaprobe II with Al Kα X-ray radiation, and XPS peaks were calibrated to the C 1s peak at 284.8 eV. The quantification analyses for HEAs by Inductively coupled plasma with an optical emission spectrometer (ICP-OES) were measured using an Agilent 5110 and analyzed data using Agilent ICP Expert software.

Synchrotron X-ray Analytical Techniques

Synchrotron X-ray pair distribution function (PDF) and wide-angle X-ray scattering (WAXS) were measured using a wavelength of 0.24152 Å on beamline 28-ID-1 at the National Synchrotron Light Source (NSLS-II), Brookhaven National Laboratory. The measurements were conducted at an X-ray energy of 74.4 keV, and the data were processed and reduced using the standard data reduction protocols employed, including pyFAI[2] for azimuthal integration and PDFgetX3[3]for creation of the PDFs. All samples were dropcast onto Kapton and dried before measurement. To

minimize interference from the PDF peaks of the Kapton tube support at 1.38 Å and 2.42 Å, background removal was performed using the PDFgetx3gui software with diffraction data collected from the empty Kapton, DI water, and ethanol. The parameters used for this process were Qmax = 19.7, $Qmax_{inst}$ = 21.60, and rpoly = 0.42. After that, the PDF data was fitted and analyzed to assess the functionality of local structures, interfaces, chemical bonds, and component-based phases using *PDFgui*. A Q_{damp} and beamline instrument parametrization were performed by fitting the NIST LaB_6 standard at the BNL NSLS-II to ensure high reliability in the simulations. Simulations optimized using the *PDFgui* model for cubic structure yield fitting values for the synthesized HEAs in the 0.98 to 30 Å range. Additionally, the lattice constants for the HEAs used in the modeling were calculated based on the XRD results: Au 9 at.% (4.07 Å), Au 15 at.% (4.07 Å), Au 31 at.% (4.077 Å), and Au 27 at.% (4.078 Å), respectively. The experimental PDF was calculated atomic pair distribution function, denoted *G*(*r*), and is the truncated Fourier transform of the total scattering structure function *S*(*Q*) using,

$$G(r) = \frac{2}{\pi}\int_0^{\infty} Q[S(Q) - 1]sin(Qr)\,dQ, \quad (1)$$

Where *Q* is the magnitude of the scattering impulse, *S*(*Q*) is extracted from the Bragg and diffuse components of X-ray, neutron, or electron powder diffraction intensity. Additionally, for macroscopic scatterers, *G*(*r*) can be derived from the known structural model as follows,

$$G(r) = 4\pi r[\rho(r) - \rho_0] \quad (2)$$

$$\rho(r) = \frac{1}{4\pi r^2}\sum_i \sum_{j\neq i} \frac{b_i b_j}{<b>^2}\delta(r - r_{ij}) \quad (3)$$

ρ(r) is the atomic pair density and ρ_0 is the atomic number density, which represents mean weighted density of neighbor atoms at distance *r* from an atom at the origin. b_i is the scattering factor of atom (*i*), <*b*> is the average scattering factor and r_{ij} is the separation between atoms *i* and

j. Details of the experimental PDF determination have been reported elsewhere[4,5].

Electrochemical measurements

A potentiostat (Pine research, Wavedriver 100) with a three-electrode system was used to perform electrochemical measurements of working electrode activity at ambient temperature (~25 °C) in acidic electrolytes (0.5M H_2SO_4). The working electrode was prepared by sonicating a mixture solution containing 1 mg of HEAs, 3 mg of mixed of supporting materials (Vulcanized Carbon), 50 μL Nafion, and 300 μL IPA to make the electrocatalyst ink. Subsequently, 10 μL of ink solution was drop-cast onto an L-type glassy carbon with an active area 0.07 cm^2. All working electrodes were prepared in the same method. All the potentials *vs.* Ag/AgCl reference values were calibrated to a reversible hydrogen electrode (RHE) using the Nernst equation as below:

$$E_{\mathrm{RHE}} = E_{\mathrm{Ag/AgCl}} + (0.0591 \times pH) + 0.197\ \mathrm{V} \tag{4}$$

where E_{RHE} is the converted potential of the *vs*. the reference electrode, $\mathrm{E}_{\mathrm{Ag/AgCl}}$ is the experimentally measured potential and 0.197 is the standard potential of Ag/AgCl. All electrochemical measurements were collected using iR compensation. The HER activity of AuPdFeCoNi HEAs were tested by linear sweep voltammetry (LSV) in the range of 0 V to -1.5 V *vs.* RHE at a rate of 5 mV/s with collected data up to ~100 mA/cm^2. To determine the Tafel slope and mass activity, polarization curves were analyzed from LSV plot, and the mass values were obtained by ICP-OES. The oxidation-reduction potential was evaluated by cyclic voltammetry (CV) at 200 mV/s. For durability comparison, the HEAs were tested using chronopotentiometry (CP) with the electrolyte replaced every 48 hours to minimize pH effects, as hydrogen evolution can lead to a gradual increase in pH due to the consumption of hydrogen ions.

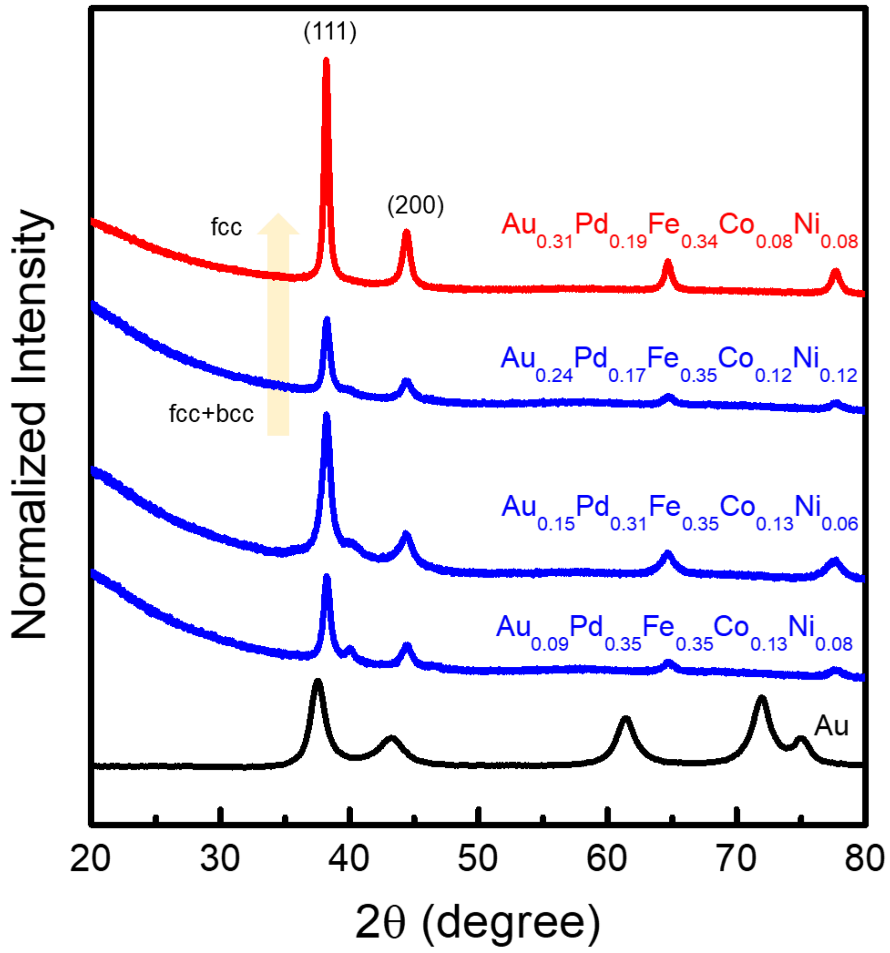

Figure S1 XRD pattern of AuPdFeCoNi HEAs in the range of 2θ = 20-80°.

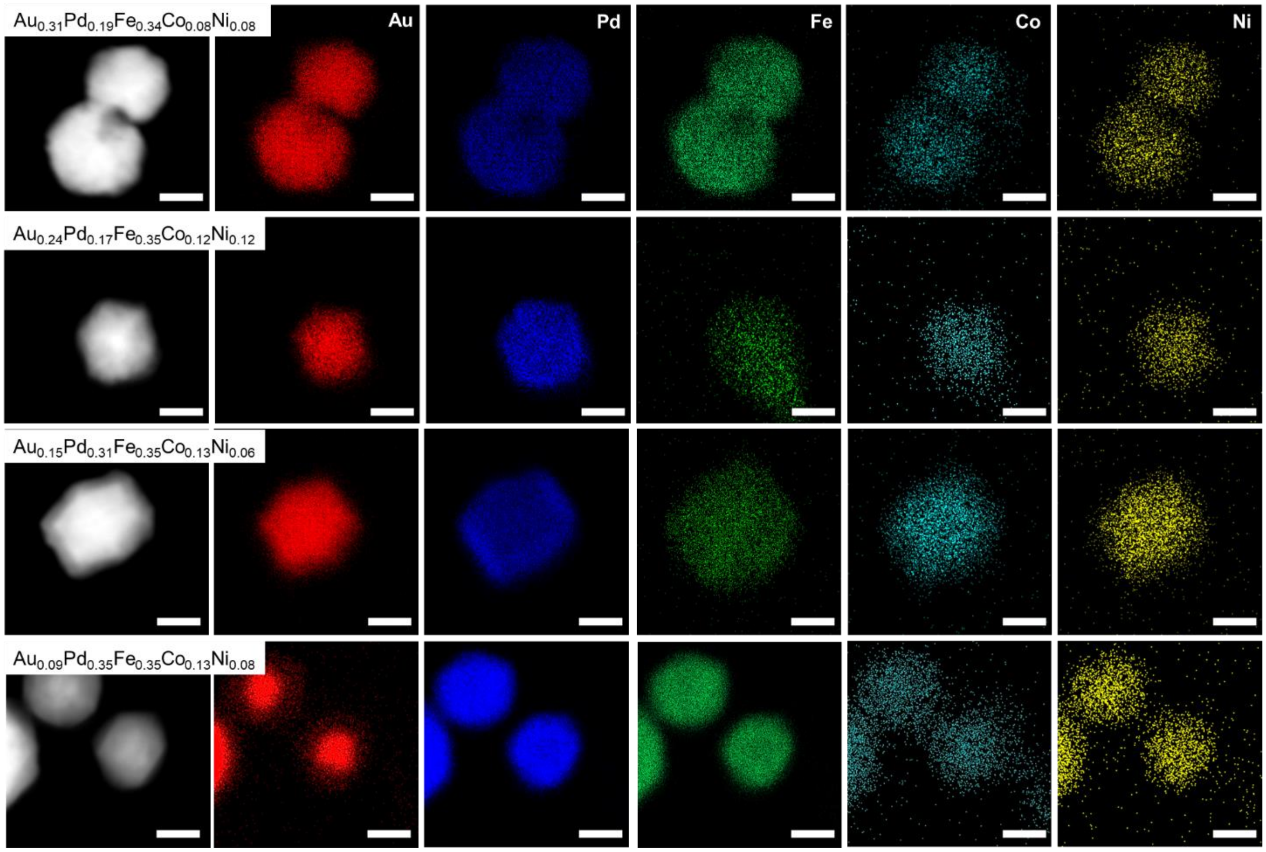

Figure S2 DF-STEM image of the HEAs with different Au content and the corresponding EDS map showing each element : Au (red), Pd (blue), Fe (green), Co(cyan), and Ni (yellow), (scale bar: 50nm).

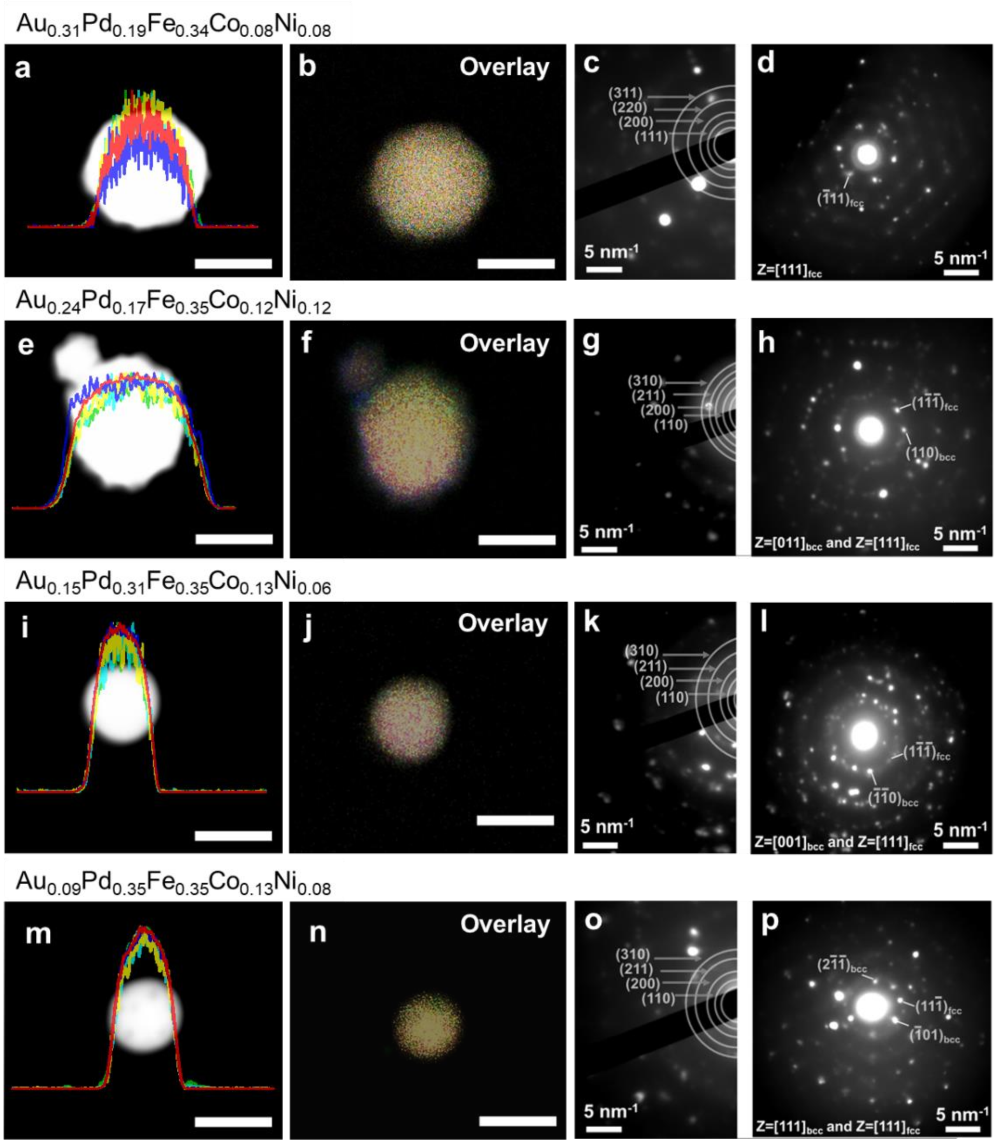

Figure S3. Characterization of HEA nanoparticles with different Au contents. (a-d) 31 at.% Au: (a) STEM image with line scan profile, (b) composite EDS map of the five metals, (c) SAED ring pattern, (d) SAED spot pattern. (e-h) 24 at.% Au: (e) STEM image with line scan profile, (f) composite EDS map of the five metals, (g) SAED ring pattern, (h) SAED spot pattern. (i-l) 15 at.% Au: (i) STEM image with line scan profile, (j) composite EDS map of the five metals, (k) SAED ring pattern, (l) SAED spot pattern. (m-p) 9 at.% Au: (m) STEM image with line scan profile, (n) composite EDS map of the five metals, (o) SAED ring pattern, (p) SAED spot pattern (Five metals false colored as: Au (red), Pd (blue), Fe (green), Co (cyan), and Ni (yellow), scale bar 200nm).

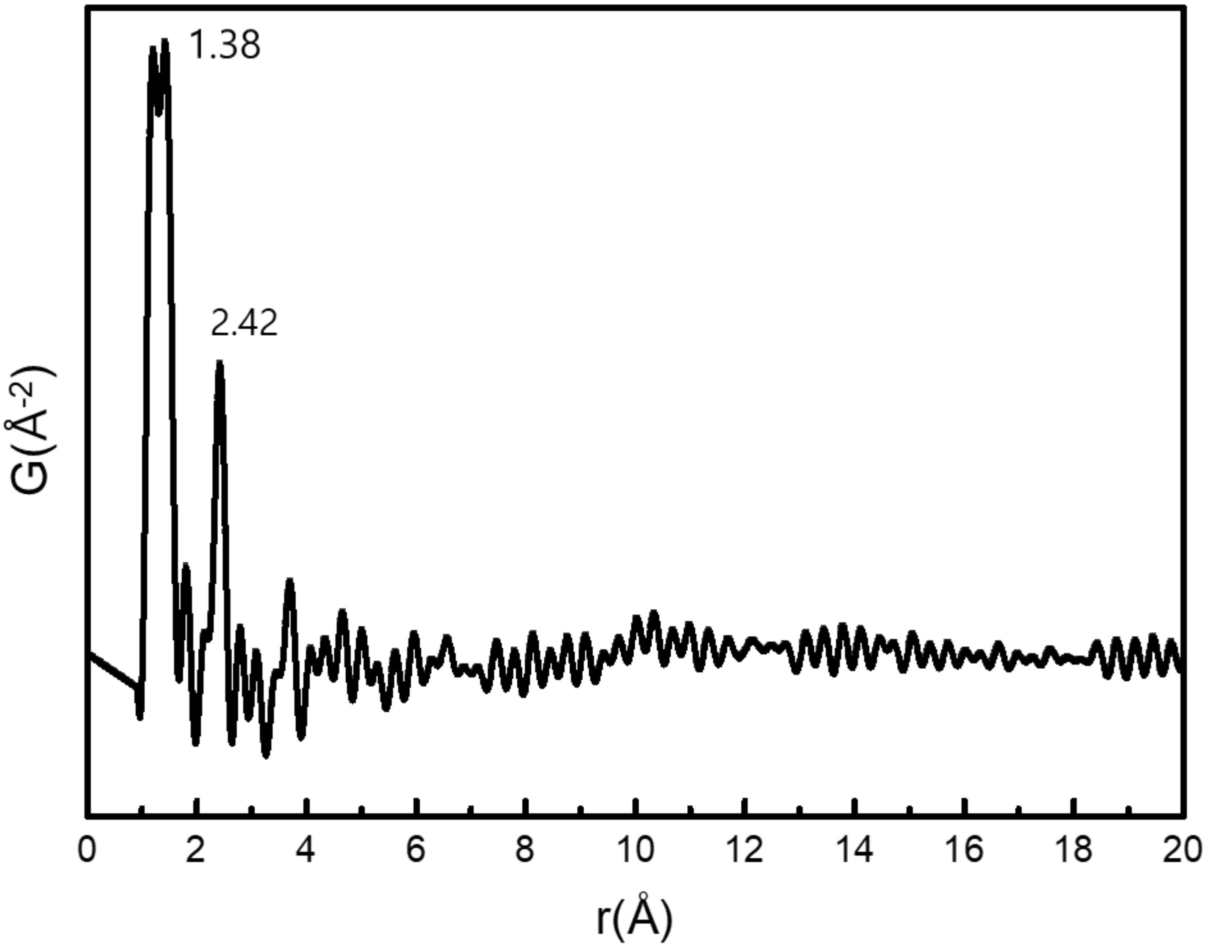

Figure S4 Pair distribution function (PDF) data for the support baseline using Kapton tube.

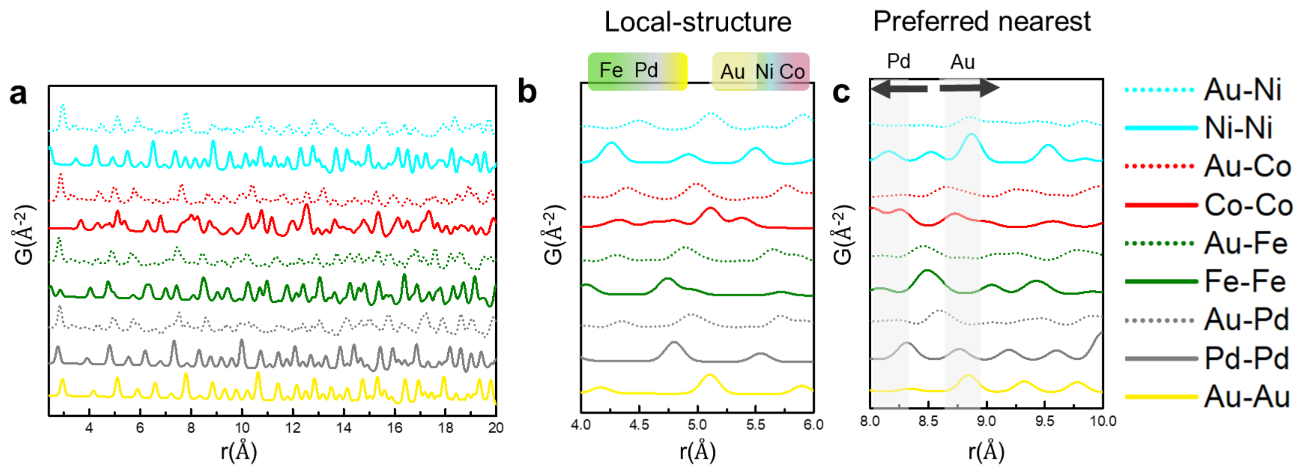

Figure S5 Local structure characterization. (a) Comparison of the observed *PDFgui* simulation model data at a scattering range of 2.8 to 20 Å. (b) Enlarged views of the PDF profiles around the peaks at r ≈ 5.0 Å, highlighting certain nearest-neighbor pairs. (c) Enlarged views of the PDF profiles around the peaks at r ≈ 9.0 Å, highlighting certain nearest-neighbor pairs.

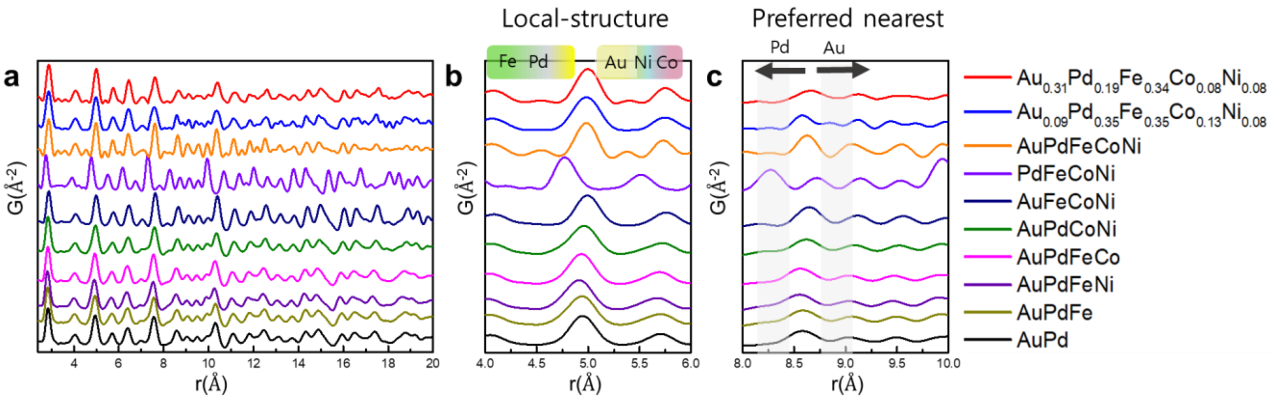

Figure S6 Local structure characterization of Au-based bimetallic-ternary-HEAs systems. (a) Comparison of the observed PDF experiment data at a scattering range of 2.8 to 20 Å. (b) Enlarged views of the PDF profiles around the peaks at r ≈ 5.0 Å, highlighting certain nearest-neighbor pairs. (c) Enlarged views of the PDF profiles around the peaks at r ≈ 8.6 Å, highlighting certain nearest-neighbor pairs.

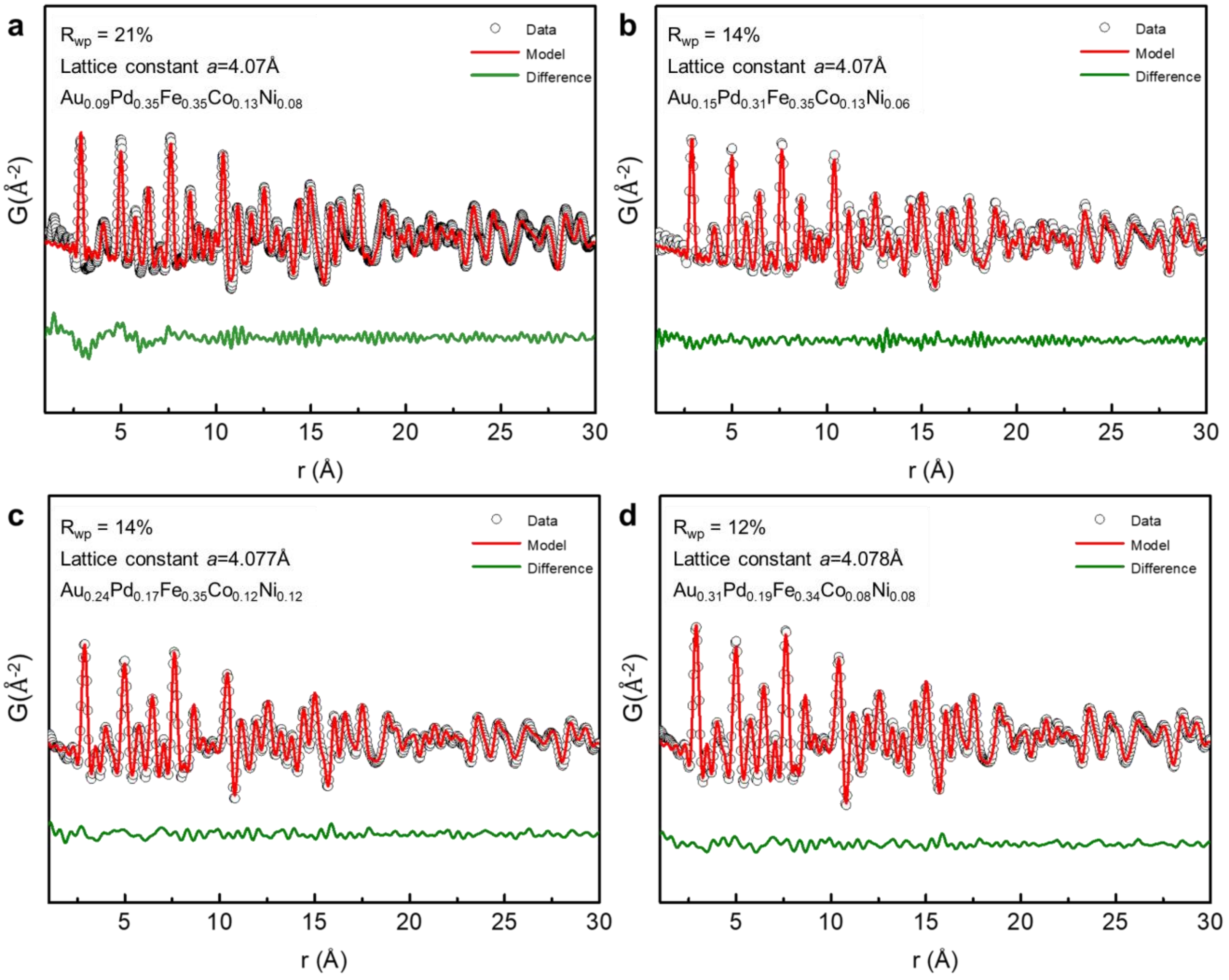

Figure S7 The experimental PDF and simulated PDF data for (a) 9 at.% Au, R_{wp} =21%, (b) 15 at.% Au, R_{wp} =14%, (c) 24 at.% Au, R_{wp} =14%, and (d) 31 at.% Au, R_{wp} =12%.

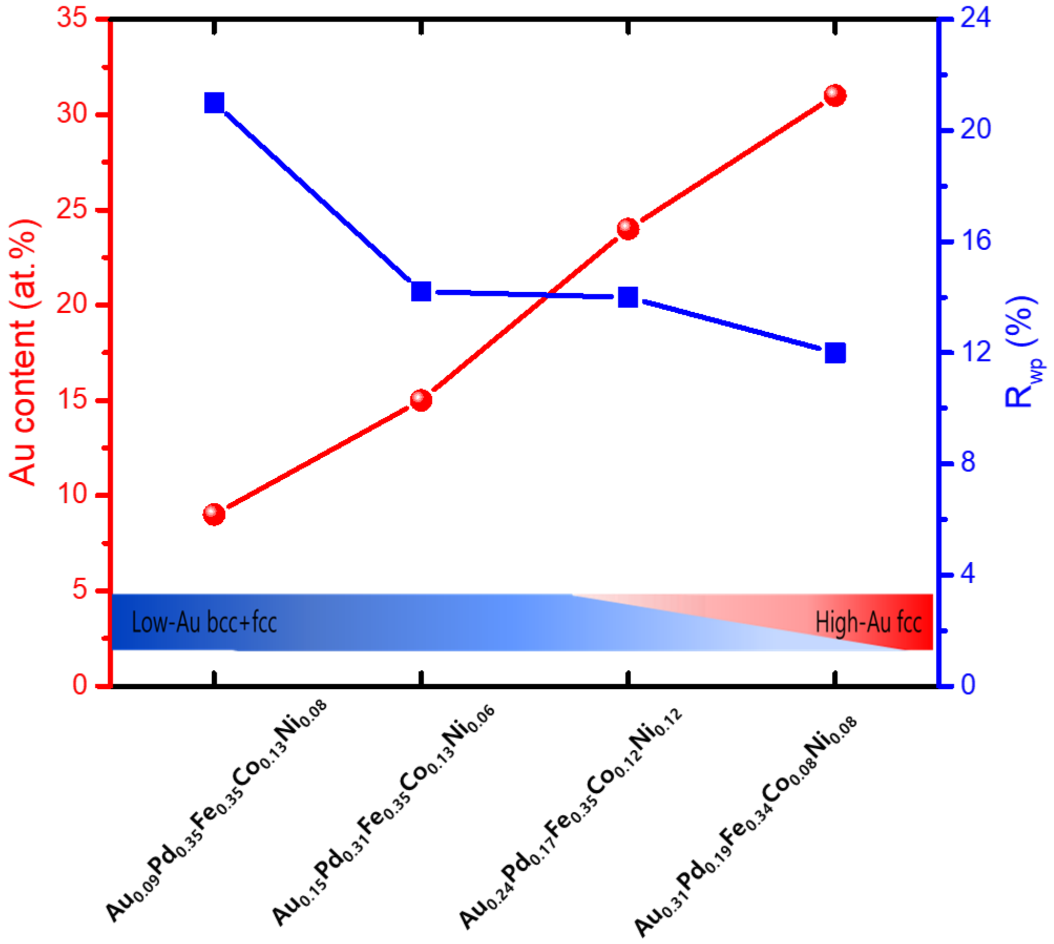

Figure S8 ICP-OES data for Au content (at.%) (left y-axis) and the Rwp values from PDF analysis (right y-axis).

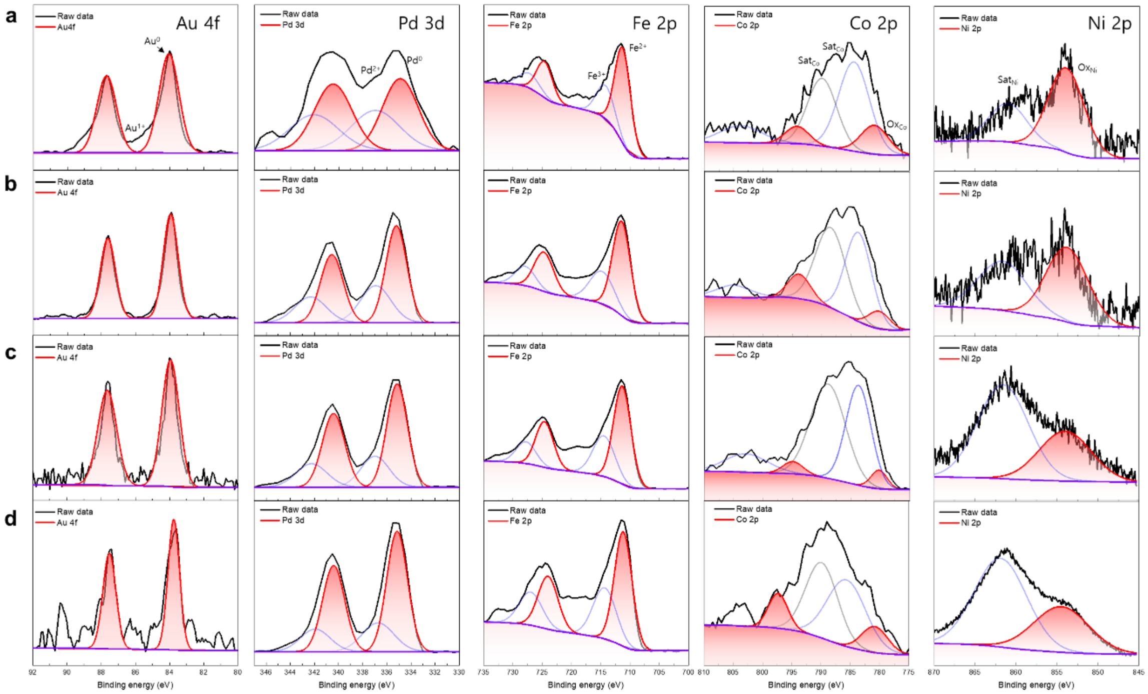

Figure S9 High-resolution XPS spectra of AuPdFeCoNi HEAs. (a) Au 31 at.%, (b) Au 24 at.%, (c) Au 15 at.%, and (d) Au 9 at.%.

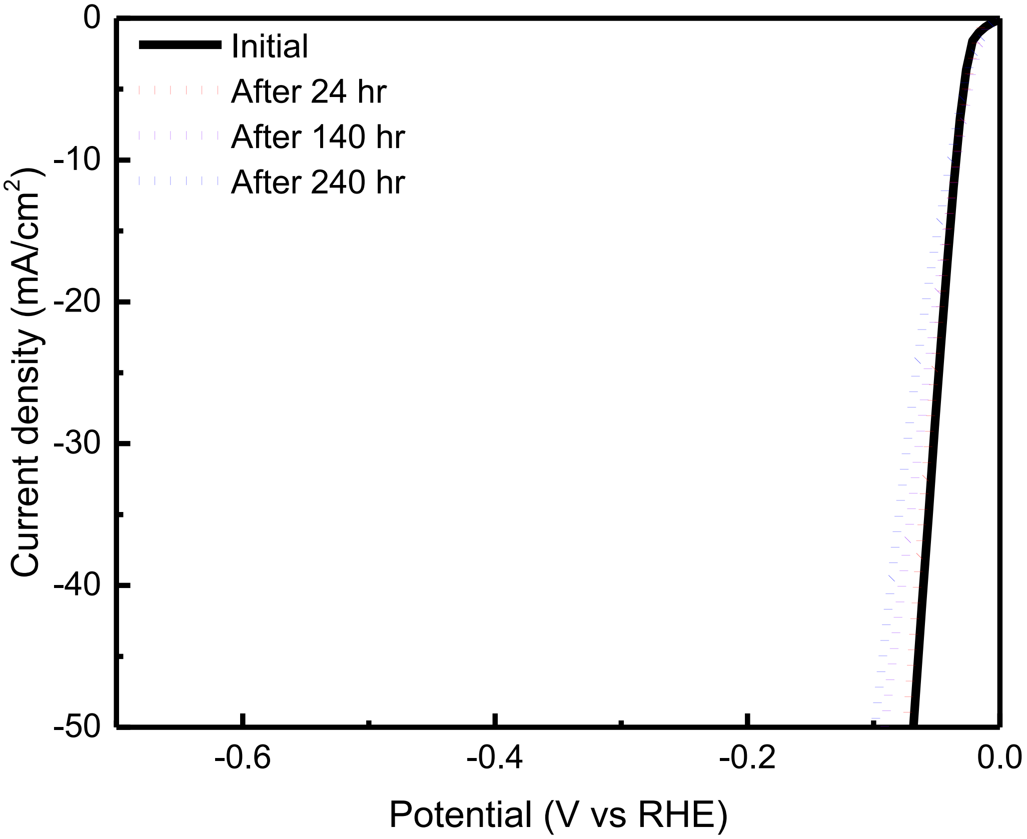

Figure S10. HER polarization curves of the as-synthesized HEAs with 15 at.% Au after stability testing: Initial (black line), 24hr (red dot), 140hr (purple dot), and 240hr (blue dot) at 10mA/cm^2.

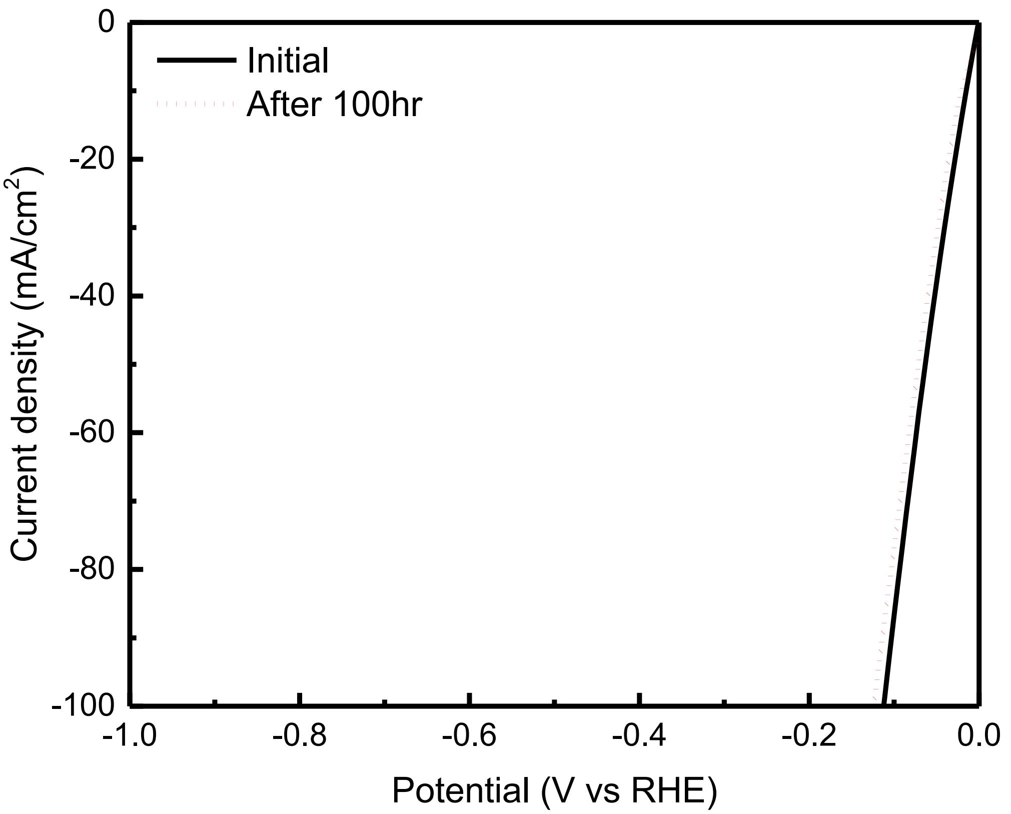

Figure S11. HER polarization curves of the as-synthesized HEAs after stability testing: Initial (black line), 100hr (red dot at 100mA/cm^2).

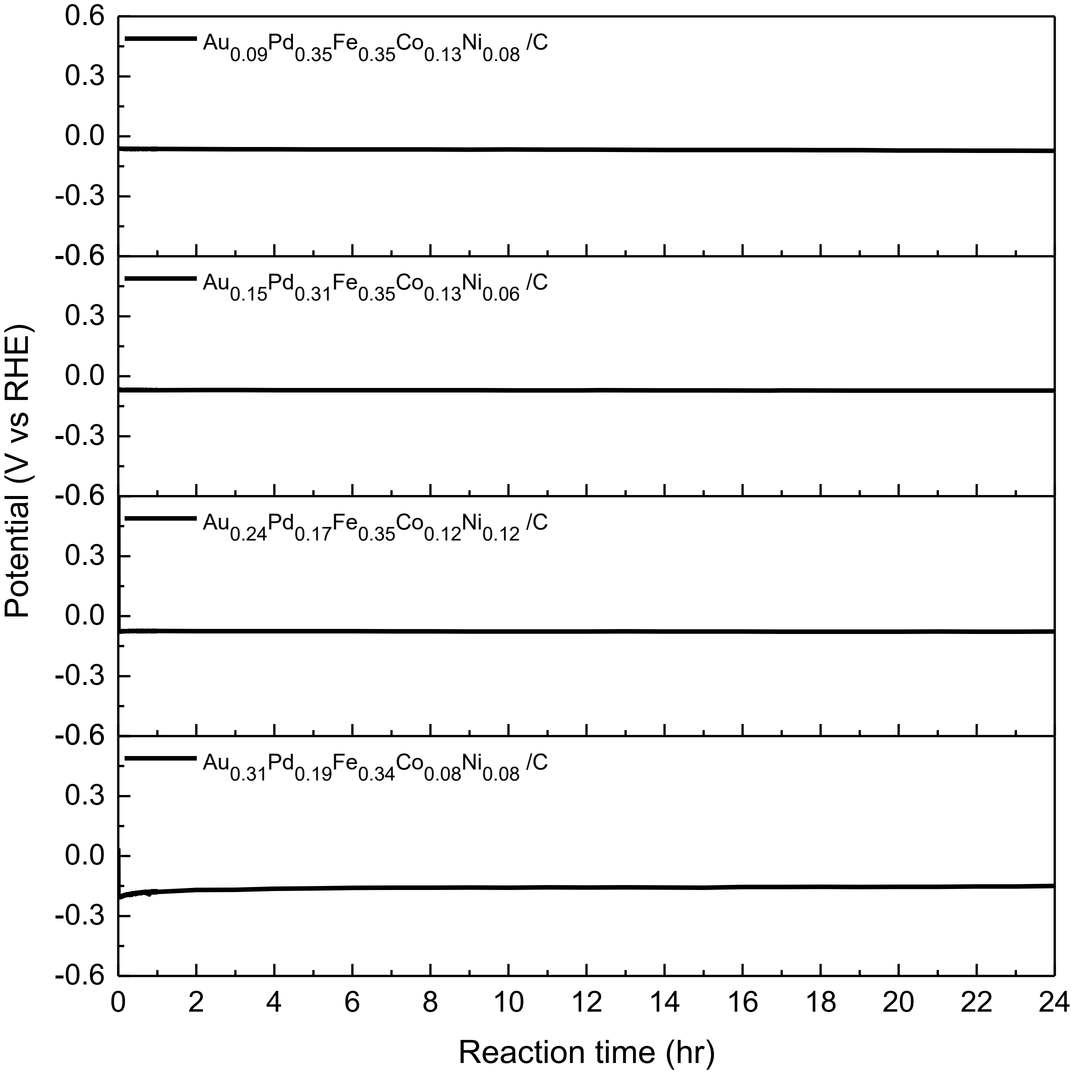

Figure S12. Long-term stability of AuPdFeCoNi HEAs for 24hr at $10mA/cm^2$.

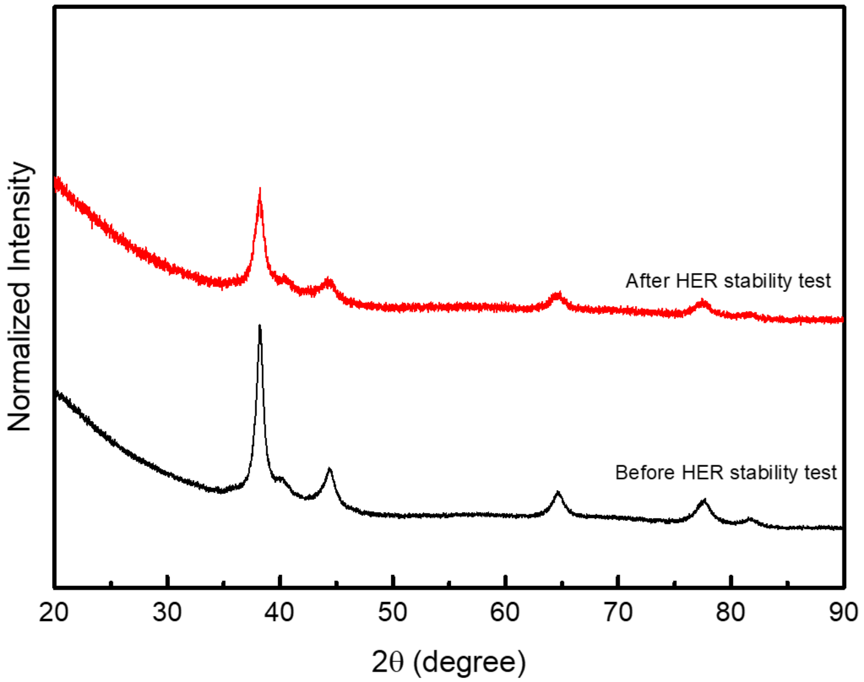

Figure S13. XRD patterns of as-synthesized HEAs with 15 at.% Au after HER stability test.

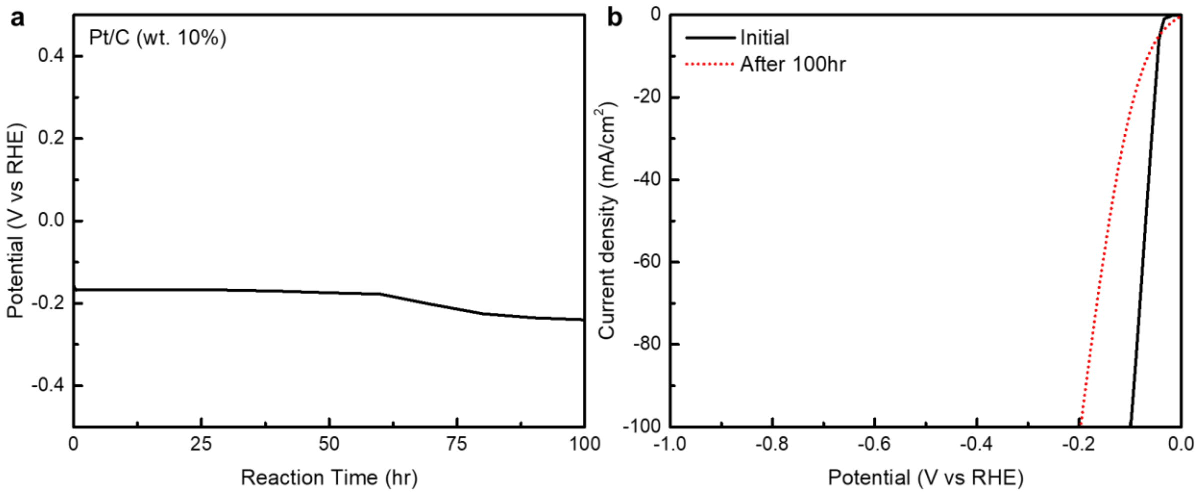

Figure S14. (a) Long-term stability of Pt/C (10 wt. %) for 100hr at 100mA/cm^2, (b) HER polarization curves of the Pt/C after stability testing: Initial (black line), 100hr (red dot at 100mA/cm^2).

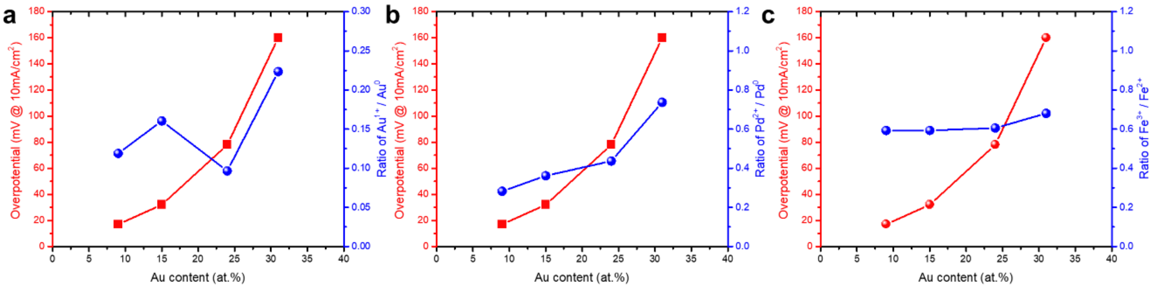

Figure S15. Electrochemical HER activity (left y-axis) and the ratio of Au 4f, Pd 3d, and Fe 2p oxidation state (right y-axis) versus Au contents.

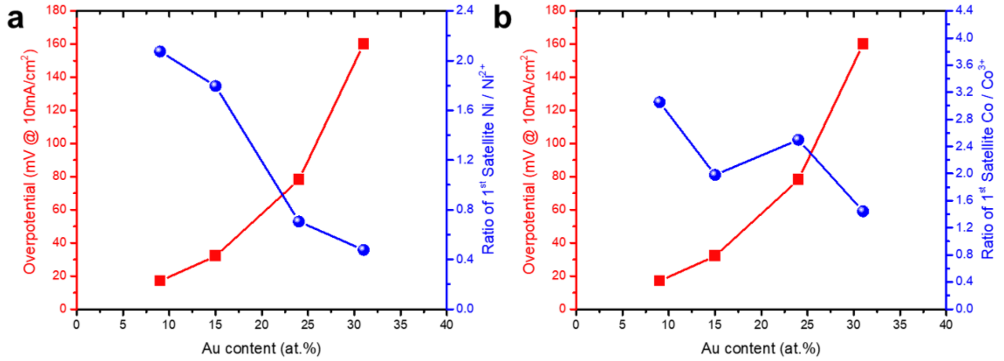

Figure S16. Electrochemical HER activity (left y-axis) and the ratio of Ni 2p and Co 2p oxidation state (right y-axis) versus Au contents.

Table S1. Quantitative ICP-OES and XPS data for AuPdFeCoNi HEAs

Atomic percentage from ICP-OES					Atomic percentage from XPS				
	HEA-1	HEA-2	HEA-3	HEA-4		HEA-1	HEA-2	HEA-3	HEA-4
Au (%)	9.28	15.36	24.01	30.92	Au (%)	10.7	15.4	22.0	32.3
Pd (%)	34.80	30.89	17.06	19.03	Pd (%)	31.9	26.7	18.4	25.4
Fe (%)	34.93	34.79	34.95	34.05	Fe (%)	33.2	33.8	32.6	27.7
Ni (%)	7.92	5.87	11.74	7.67	Ni (%)	10.7	9.8	12.3	7.7
Co (%)	13.07	13.09	12.24	8.33	Co (%)	13.5	14.3	14.7	6.9

Table S2. Chemical shift phenomenon for Au, Pd, Fe, Co, and Ni chemical states

**Chemical binding energy (BE, eV)**								
**eV**	HEA (Au 9 at.%)		HEA (Au 15 at.%)		HEA (Au 24 at.%)		HEA (Au 31 at.%)	
	Data Center	Deconvolution of B.E	Data Center	Deconvolution of B.E	Data Center	Deconvolution of B.E	Data Center	Deconvolution of B.E
Au 4f	83.6	83.6	84.0	83.8	84.0	84.0	84.0	84.0
Pd 3d	335.5	335.1	335.5	335.1	335.5	335.2	335.5	335.1
Fe 2p	711.5	711.2	711.5	711.4	711.5	711.5	711.5	711.5
Co 2p	789.0	780.6	787.5	780.4	787.5	780.5	786.5	780.5
Ni 2p	854.8	854.5	854.8	854.1	854.1	854.1	854.1	854.0

Table S3. Economic cost for fabrication of HEAs by using raw materials

Precursor	Price per unit	Wt. %	Total price
$AuCl_3$	$ 218 / g	2.69	$ 5.86
$PdCl_2$	$ 214 / g	5.56	$ 11.98
$FeCl_2H_8O_4$	$ 3.98 / g	6.28	$ 0.25
$CoCl_2H_{12}O_6$	$ 1.26 / g	1.08	$ 0.01
$NiCl_2$	$ 11.22 / g	2.33	$ 0.26
Total			$ 18.36

Table S4. Comparison of the overpotential, stability and the mass activity for HER[6-26]

HEAs	Overpotential (@ 10mA/cm^2)	Stability	Mass activity (A/mg$_{PGM}$)	Ref.
AuPdFeNiCo	17 mV	100 hr (@100mA/cm^2)	50 (@150mV)	This work
PdPtRuRhAu	70.07 mV	90 hr (@100mA/cm^2)	-	6 (Adv. Mater. 2025)
PdMoGaInNi	13 mV	12hr (@10mA/cm^2)	-	7 (ACS Catal. 2022)
PdMoInFeCoNi	40 mV	-	-	7 (ACS Catal. 2022)
AlNiCoIrMo	18.5 mV	48hr (@10mA/cm^2)	0.115 (@150mV)	8 (Small, 2019)
CoCrFeNi/SUS	470 mV	12.5hr (@5mA/cm^2)	-	9 (J. Phys. Chem. C 2021)
RuRhPdAgOsIrPtAu	60 mV	-	-	10 (J. Am. Chem. Soc.2022)
NiFeMoCoCr	172 mV	8hr (@100mA/cm^2)	-	11 (Electrochim. Acta. 2018)
CoFeNiMnCr	403 mV	10hr (@10mA/cm^2)	-	12 (Mater. Today Commun.2024)
IrPdPtRhRu	33 mV	-	-	13 (Chem. Sci., 2020)
PdPtCuNiP	62 mV	90hr (@10mA/cm^2)	-	14 (Adv. Funct. Mater. 2021)
FeNiCoRhPt	27 mV	100hr (@30mA/cm^2)	116.1 (@150mV)	15 (J. Am. Chem. Soc. 2021)
PtFeCoNiCu	20 mV	80hr (@10mA/cm^2)	-	16 (Nat. Commun. 2024)
PtCoNiRuIr/C	18 mV	120hr (@50mA/cm^2)	11.8 (@70mV)	17 (ACS Nano 2024)
(MoWV)Se2	103 mV	120hr (@10mA/cm^2)	-	18 (ACS Nano 2023)
FeRuPtNiCoPd/GO	49 mV	30hr (@10/20mA/cm^2)	29.35 (@100mV)	19 (J. Mater. Chem. A, 2025)
CoCrFeNiAl	125 mV	12hr (@10mA/cm^2)	-	20 (J. Materiomics 2020)
FeCoNiMoPtRu-QDs	11 mV	20hr (@50mA/cm^2)	9 (@150mV)	21 (Energy Environ. Sci, 2024)
PtIrRuRhPd	20 mV	55hr (@10mA/cm^2)	-	22 (J. Mater. Chem. A, 2024)
FeCoNiCrPt	46 mV	24hr (@10mA/cm^2)	-	23 (Energy Environ. Sci, 2024)
PdRhMoFeMn	6 mV	20hr (@10mA/cm^2)	35.7 (@50mV)	16 (Nat. Commun. 2024)
Pd@PtRuFeCoNi	41.3 mV	30hr (@15,000 cycles)	-	24 (Sci. Adv. 2024)
PdPtRhIrRu	30 mV	20hr (@100mA/cm^2)	-	25 (Sci. Adv. 2023)

References

(1) Jeong, S.; Branco, A. J.; Bollen, S. W.; Sullivan, C. S.; Ross, M. B. Universal PH Electrocatalytic Hydrogen Evolution with Au-Based High Entropy Alloys. *Nanoscale* **2024**, 11530–11537. https://doi.org/10.1039/d4nr01538j.

(2) Kieffer, J.; Karkoulis, D. PyFAI, a Versatile Library for Azimuthal Regrouping. *Journal of Physics: Conference Series* **2013**, *425*. https://doi.org/10.1088/1742-6596/425/20/202012.

(3) Juhás, P.; Davis, T.; Farrow, C. L.; Billinge, S. J. L. PDFgetX3 : A Rapid and Highly Automatable Program for Processing Powder Diffraction Data into Total Scattering Pair Distribution Functions. *Journal of Applied Crystallography* **2013**, *46* (2), 560–566. https://doi.org/10.1107/S0021889813005190.

(4) Farrow, C. L.; Juhas, P.; Liu, J. W.; Bryndin, D.; Boin, E. S.; Bloch, J.; Proffen, T.; Billinge, S. J. L. PDFfit2 and PDFgui: Computer Programs for Studying Nanostructure in Crystals. *Journal of Physics Condensed Matter* **2007**, *19* (33). https://doi.org/10.1088/0953-8984/19/33/335219.

(5) Billinge, S. J. L. The Rise of the X-Ray Atomic Pair Distribution Function Method: A Series of Fortunate Events. *Philosophical Transactions of the Royal Society A: Mathematical, Physical and Engineering Sciences* **2019**, *377* (2147). https://doi.org/10.1098/rsta.2018.0413.

(6) Wang, Q.; Qin, Y.; Xie, J.; Kong, Y.; Sun, Q.; Wei, Z.; Zhao, S. Size-Controllable High-Entropy Alloys Toward Stable Hydrogen Production at Industrial-Scale Current Densities. *Advanced Materials* **2025**, *37* (10). https://doi.org/10.1002/adma.202420173.

(7) Fu, X.; Zhang, J.; Zhan, S.; Xia, F.; Wang, C.; Ma, D.; Yue, Q.; Wu, J.; Kang, Y. High-Entropy Alloy Nanosheets for Fine-Tuning Hydrogen Evolution. *ACS Catal* **2022**, *12* (19), 11955–11959. https://doi.org/10.1021/acscatal.2c02778.

(8) Jin, Z.; Lv, J.; Jia, H.; Liu, W.; Li, H.; Chen, Z.; Lin, X.; Xie, G.; Liu, X.; Sun, S.; Qiu, H. J. Nanoporous Al-Ni-Co-Ir-Mo High-Entropy Alloy for Record-High Water Splitting Activity in Acidic Environments. *Small* **2019**, *15* (47). https://doi.org/10.1002/smll.201904180.

(9) McKay, F.; Fang, Y.; Kizilkaya, O.; Singh, P.; Johnson, D. D.; Roy, A.; Young, D. P.; Sprunger, P. T.; Flake, J. C.; Shelton, W. A.; Xu, Y. CoCrFeNi High-Entropy Alloy as an Enhanced Hydrogen Evolution Catalyst in an Acidic Solution. *Journal of Physical Chemistry C* **2021**, *125* (31), 17008–17018. https://doi.org/10.1021/acs.jpcc.1c03646.

(10) Wu, D.; Kusada, K.; Nanba, Y.; Koyama, M.; Yamamoto, T.; Toriyama, T.; Matsumura, S.; Seo, O.; Gueye, I.; Kim, J.; Rosantha Kumara, L. S.; Sakata, O.; Kawaguchi, S.; Kubota, Y.; Kitagawa, H. Noble-Metal High-Entropy-Alloy Nanoparticles: Atomic-Level Insight into the Electronic Structure. *J Am Chem Soc* **2022**, *144* (8), 3365–3369. https://doi.org/10.1021/jacs.1c13616.

(11) Zhang, G.; Ming, K.; Kang, J.; Huang, Q.; Zhang, Z.; Zheng, X.; Bi, X. High Entropy Alloy as a Highly Active and Stable Electrocatalyst for Hydrogen Evolution Reaction. *Electrochim Acta* **2018**, *279*, 19–23. https://doi.org/10.1016/j.electacta.2018.05.035.

(12) Ljubec Božiček, B.; Arah, B.; Kušter, M.; Naglič, I.; Markoli, B.; Ponikvar-Svet, M.; Einfalt, L.; Čeh, M.; Alcantara Marinho, B. Electrocatalytic Trends of Different Cantor Entropy Alloys for Alkaline and Acidic Hydrogen-Evolution Reactions. *Mater Today Commun* **2024**, *41*. https://doi.org/10.1016/j.mtcomm.2024.110876.

(13) Wu, D.; Kusada, K.; Yamamoto, T.; Toriyama, T.; Matsumura, S.; Gueye, I.; Seo, O.; Kim, J.; Hiroi, S.; Sakata, O.; Kawaguchi, S.; Kubota, Y.; Kitagawa, H. On the Electronic Structure and Hydrogen Evolution Reaction Activity of Platinum Group Metal-Based High-Entropy-Alloy Nanoparticles. *Chem Sci* **2020**, *11* (47), 12731–12736. https://doi.org/10.1039/d0sc02351e.

(14) Wang, D.; Li, H.; Du, N.; Hou, W. Single Platinum Atoms Immobilized on Monolayer Tungsten Trioxide Nanosheets as an Efficient Electrocatalyst for Hydrogen Evolution Reaction. *Adv Funct Mater* **2021**, *31* (23). https://doi.org/10.1002/adfm.202009770.

(15) Feng, G.; Ning, F.; Song, J.; Shang, H.; Zhang, K.; Ding, Z.; Gao, P.; Chu, W.; Xia, D. Sub-2 Nm Ultrasmall High-Entropy Alloy Nanoparticles for Extremely Superior Electrocatalytic Hydrogen Evolution. *J Am Chem Soc* **2021**, *143* (41), 17117–17127. https://doi.org/10.1021/jacs.1c07643.

(16) Chen, Z. W.; Li, J.; Ou, P.; Huang, J. E.; Wen, Z.; Chen, L. X.; Yao, X.; Cai, G. M.; Yang, C. C.; Singh, C. V.; Jiang, Q. Unusual Sabatier Principle on High Entropy Alloy Catalysts for Hydrogen Evolution Reactions. *Nat Commun* **2024**, *15* (1). https://doi.org/10.1038/s41467-023-44261-4.

(17) Cui, X.; Liu, Y.; Wang, X.; Tian, X.; Wang, Y.; Zhang, G.; Liu, T.; Ding, J.; Hu, W.; Chen, Y. Rapid High-Temperature Liquid Shock Synthesis of High-Entropy Alloys for Hydrogen Evolution Reaction. *ACS Nano* **2024**, *18* (4), 2948–2957. https://doi.org/10.1021/acsnano.3c07703.

(18) Kwon, I. S.; Lee, S. J.; Kim, J. Y.; Kwak, I. H.; Zewdie, G. M.; Yoo, S. J.; Kim, J. G.; Lee, K. S.; Park, J.; Kang, H. S. Composition-Tuned (MoWV)Se2 Ternary Alloy Nanosheets as Excellent Hydrogen Evolution Reaction Electrocatalysts. *ACS Nano* **2023**, *17* (3), 2968–2979. https://doi.org/10.1021/acsnano.2c11528.

(19) Lee, Y.; Theerthagiri, J.; Limphirat, W.; Periyasamy, G.; Jeong, G. H.; Kheawhom, S.; Tang, Y.; Choi, M. Y. Pulsed Laser-Patterned High-Entropy Single-Atomic Sites and Alloy Coordinated Graphene Oxide for PH-Universal Water Electrolysis. *J Mater Chem A Mater* **2025**, *13* (13), 9073–9087. https://doi.org/10.1039/d5ta00117j.

(20) Ma, P.; Zhao, M.; Zhang, L.; Wang, H.; Gu, J.; Sun, Y.; Ji, W.; Fu, Z. Self- Supported High-Entropy Alloy Electrocatalyst for Highly Efficient H2 Evolution in Acid Condition. *Journal of Materiomics* **2020**, *6* (4), 736–742. https://doi.org/10.1016/j.jmat.2020.06.001.

(21) Zhao, H.; Liu, M.; Wang, Q.; Li, Y. Z.; Chen, Y.; Zhu, Y.; Yue, Z.; Li, J.; Wang, G.; Zou, Z.; Cheng, Q.; Yang, H. Strong Transboundary Electron Transfer of High-Entropy Quantum-Dots Driving Rapid Hydrogen Evolution Kinetics. *Energy Environ Sci* **2024**, *17* (18), 6594–6605. https://doi.org/10.1039/d4ee01825g.

(22) Su, K.; Yang, S.; Zhu, Y.; Liang, Y.; Tang, Y.; Qiu, X. High-Entropy Alloy Nanocages with Highly Ordered {100} Facets and Ultrathin Features for Water Splitting in an Acidic Medium. *J Mater Chem A Mater* **2024**, *12* (26), 16043–16051. https://doi.org/10.1039/d4ta02556c.

(23) Yang, Y.; Jia, Z.; Wang, Q.; Liu, Y.; Sun, L.; Sun, B.; Kuang, J.; Dai, S.; He, J.; Liu, S.; Duan, L.; Tang, H.; Zhang, L. C.; Kruzic, J. J.; Lu, J.; Shen, B. Vacancy Induced Microstrain in High-Entropy Alloy Film for Sustainable Hydrogen Production under Universal PH Conditions. *Energy Environ Sci* **2024**, *17* (16), 5854–5865. https://doi.org/10.1039/d4ee01139b.

(24) Wu, C.-Y.; Hsiao, Y.-C.; Chen, Y.; Lin, K.-H.; Lee, T.-J.; Chi, C.-C.; Lin, J.-T.; Hsu, L.-C.; Tsai, H.-J.; Gao, J.-Q.; Chang, C.-W.; Kao, I.-T.; Wu, C.-Y.; Lu, Y.-R.; Pao, C.-W.; Hung, S.-F.; Lu, M.-Y.; Zhou, S.; Yang, T.-H. A Catalyst Family of High-Entropy Alloy Atomic Layers with

Square Atomic Arrangements Comprising Iron-and Platinum-Group Metals. *Sci. Adv.* **2024**, *10*, 3693. https://doi.org/10.1126/sciadv.adl3693.

(25) Liu, Y. H.; Hsieh, C. J.; Hsu, L. C.; Lin, K. H.; Hsiao, Y. C.; Chi, C. C.; Lin, J. T.; Chang, C. W.; Lin, S. C.; Wu, C. Y.; Gao, J. Q.; Pao, C. W.; Chang, Y. M.; Lu, M. Y.; Zhou, S.; Yang, T. H. Toward Controllable and Predictable Synthesis of Highentropy Alloy Nanocrystals. *Sci Adv* **2023**, *9* 12023. https://doi.org/10.1126/sciadv.adf993.
